# CXXC5 as an unmethylated CpG dinucleotide binding protein contributes to estrogen-mediated cellular proliferation

**DOI:** 10.1038/s41598-020-62912-0

**Published:** 2020-04-06

**Authors:** Gamze Ayaz, Negin Razizadeh, Pelin Yaşar, Gizem Kars, Deniz Cansen Kahraman, Özge Saatci, Özgür Şahin, Rengül Çetin-Atalay, Mesut Muyan

**Affiliations:** 1 Department of Biological Sciences, Middle East Technical University, Ankara, 06800 Turkey; 2 Enformatics Institute, Middle East Technical University, Ankara, 06800 Turkey; 3 Cansyl Laboratories, Middle East Technical University, Ankara, 06800 Turkey; 4 Drug Discovery and Biomedical Sciences, College of Pharmacy, University of South Carolina, Columbia, SC, 29208 USA; 5 Department of Molecular Biology and Genetics, Bilkent University, Ankara, 06800 Turkey; 6 Present Address: Cancer and Stem Cell Epigenetics Section, Laboratory of Cancer Biology and Genetics, Center for Cancer Research, National Cancer Institute, National Institutes of Health, Bethesda, MD 20892 USA

**Keywords:** Cell signalling, Breast cancer

## Abstract

Evidence suggests that the CXXC type zinc finger (ZF-CXXC) protein 5 (CXXC5) is a critical regulator/integrator of various signaling pathways that include the estrogen (E2)-estrogen receptor α (ERα). Due to its ZF-CXXC domain, CXXC5 is considered to be a member of the ZF-CXXC family, which binds to unmethylated CpG dinucleotides of DNA and through enzymatic activities for DNA methylation and/or chromatin modifications generates a chromatin state critical for gene expressions. Structural/functional features of CXXC5 remain largely unknown. CXXC5, suggested as transcription and/or epigenetic factor, participates in cellular proliferation, differentiation, and death. To explore the role of CXXC5 in E2-ERα mediated cellular events, we verified by generating a recombinant protein that CXXC5 is indeed an unmethylated CpG binder. We uncovered that CXXC5, although lacks a transcription activation/repression function, participates in E2-driven cellular proliferation by modulating the expression of distinct and mutual genes also regulated by E2. Furthermore, we found that the overexpression of *CXXC5*, which correlates with mRNA and protein levels of ERα, associates with poor prognosis in ER-positive breast cancer patients. Thus, CXXC5 as an unmethylated CpG binder contributes to E2-mediated gene expressions that result in the regulation of cellular proliferation and may contribute to ER-positive breast cancer progression.

## Introduction

Estrogen hormones, particularly 17β-estradiol (E2), are involved in the physiological and pathophysiological regulation of many organ and tissue functions, including breast tissue^1^. The effects of E2 in breast tissue are mediated mainly by estrogen receptor α (ERα) as a transcription factor^1^. Because of the critical role of the E2-ERα signaling in physiology and pathophysiology of breast tissue, identifying key genes regulated by E2-ERα to alter cellular phenotype could provide new prognostic tools and/or therapeutic targets for breast cancer^1^. We previously and others recently^2^ reported that *CXXC5* is an E2-ERα responsive gene^3,4^. Due to a highly conserved zinc-finger CXXC domain (ZF-CXXC), CXXC5 is considered to be a member of the functionally distinct ZF-CXXC family encompassing 12 proteins^5–7^. The ZF-CXXC domain is characterized by two conserved cysteine-rich motifs (CXXCXXC; wherein X indicates other residues) that associate with two Zn^++^ ions forming zinc-finger structures. The ZF-CXXC family proteins through the ZF-CXXC domain recognize and bind to unmethylated CpG dinucleotides with varying affinities and, in a sequence context, specificities^7^ to regulate gene expressions^5,6^.

Expressed in cells of different tissues in response to morphogenic retinoic acid^8^, multifunctional cytokine family member transforming growth factor-β^9^ and bone morphogenetic protein 4^10,11^ as well as the Wnt family of secreted glycolipoprotein Wnt3a^12–14^, CXXC5 is suggested to participate as transcription factor, co-regulator and/or epigenetic factor in a wide variety of cellular functions. These include the modulation of signal transduction, DNA damage response, cellular energy metabolism, proliferation, differentiation, angiogenesis and cell death^8–10,12,15–19^. *In vivo* experimental models further indicate that CXXC5 contributes to osteoblast differentiation, growth plate senescence, cutaneous wound healing, hair loss, and antiviral responses as well as kidney and heart development^2,12,13,20–24^. In accordance with the importance of CXXC5 in cellular events, de-regulated expressions of *CXXC5* appear to correlate with the development, and resistance to therapies, of various pathologies including cardiovascular disease, diminished ovarian reserve (DOR), Blepharophimosis Ptosis Epicantus Inversus Syndrome (BPES), Acute Myeloid Leukemia (AML), prostate and breast cancer^8,25–30^.

Although we showed that the E2-ERα signaling augments the expression of *CXXC5* and the synthesis of the encoded protein^3,4^, the role of CXXC5 in cellular events mediated by E2-ERα is unclear. To address this issue, we here initially verified by generating a full-length recombinant protein that CXXC5 is indeed an unmethylated CpG binding protein. In assessing intracellular functions in ERα-synthesizing and E2-responsive MCF7 cells derived from a breast adenocarcinoma, we found that CXXC5, although lacks a transcription activation or repression function, modifies gene expressions independently from and in collaboration with the E2-ERα signaling. This results in the modulation of E2-mediated cellular proliferation.

## Results

### **CXXC5 is an unmethylated CpG binding protein**

*CXXC5* encodes a 322 amino-acid long protein with a molecular mass of 33 kDa. Due to its ZF-CXXC domain, CXXC5 is considered to be a member of the ZF-CXXC family^5–7^. The CXXC domain specifically binds to an unmethylated CpG dinucleotide containing DNA. Consistent with this, recent structural studies clearly indicated that the CXXC domain of CXXC5, as the other members of the ZF-CXXC family, also preferentially binds to unmethylated CpG dinucleotides^7^.

**Figure 1:**
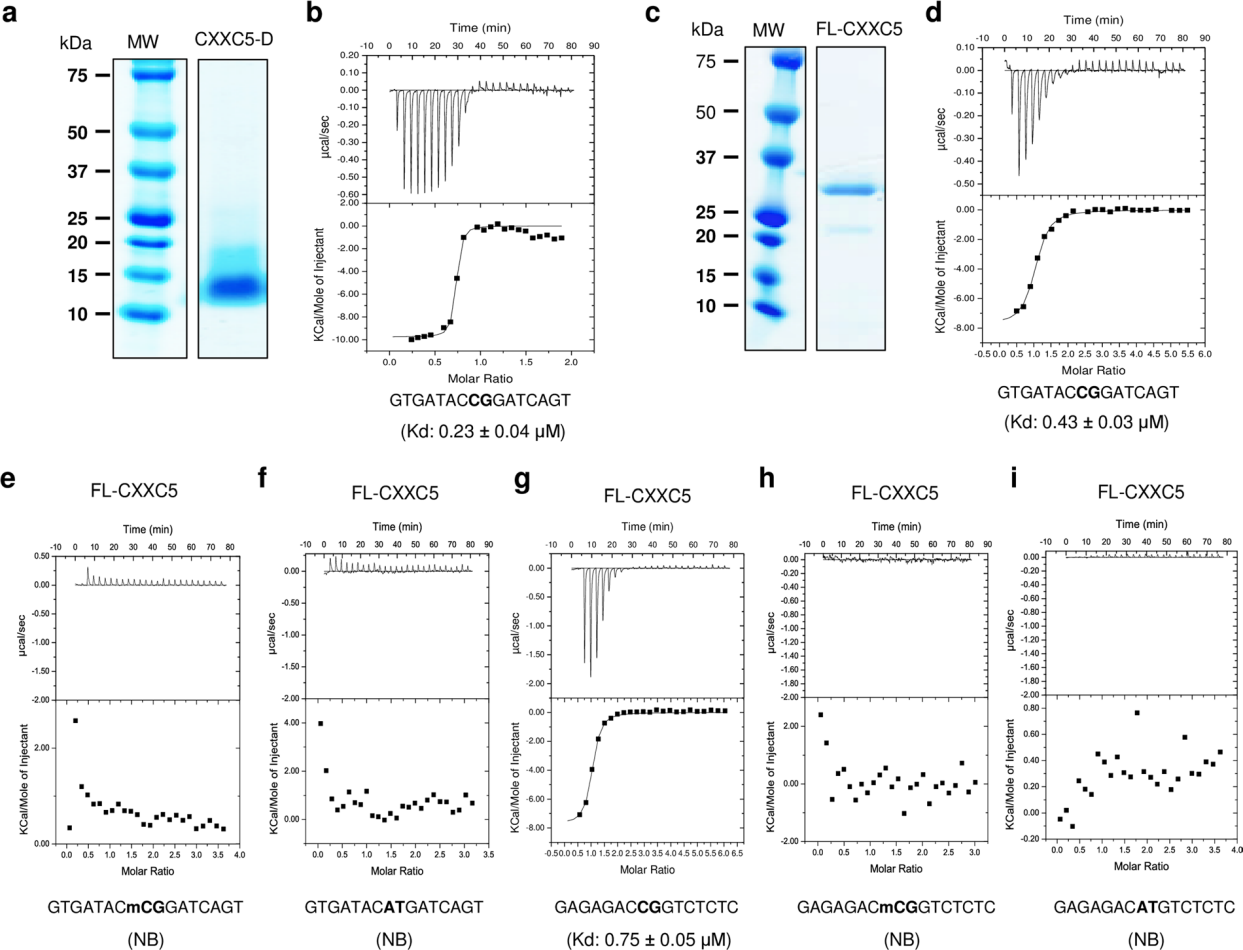
Purification and interaction with DNA of the recombinant full-length CXXC5 (FL-CXXC5) and the CXXC domain (CXXC-D) proteins. CXXC-D **(a)** and FL-CXXC5 **(c)**, expressed in bacteria and purified sequentially with ion exchange, heparin, and size-exclusion chromatographies, were loaded onto an SDS-PAGE gel (4–20% gradient) and stained with InstantBlue coomassie dye. “MW” indicates molecular masses in kDa. 10 µM CXXC-D (**b**) or FL-CXXC5 (**d**) was subjected to isothermal titration calorimetry (ITC) using a 300 µM double-stranded DNA fragment that bears a central unmethylated ***CG*** dinucleotide (5′-GTGATAC***CG***GATCAGT-3′). **(e,f)** The binding of FL-CXXC5 was also assessed with ITC using a double-stranded DNA fragment with the central ***mCG*** or ***AT*** nucleotides embedded into the same surrounding DNA sequence. To ensure that sequences surrounding the central nucleotides have no effect on the ability of FL-CXXC5 to bind to DNA, a double-stranded DNA fragment with a distinct surrounding sequence (5′-GAGAGAC***xx***GTCTCTC-3′) bearing the central ***CG*** (**G**), ***mCG*** (**h**) or ***AT*** (**i**) nucleotides was subjected to ITC. Results are the mean ± S.E. of two experiments. NB denotes no detectable binding.

Despite the structured CXXC domain located at the carboxyl-terminus of CXXC5 (250–322), our *in silico* analyses suggest that the amino-terminus of the protein is highly disordered (Supplementary Fig. S1) and lacks, as indicated previously^5–7^, any known structural motif. Since intramolecular interactions among structural regions are also critical for the functional features of a protein^31^, we wanted to assess whether or not CXXC5 as the full-length protein binds to unmethylated CpG dinucleotide containing DNA as well. To examine this issue, we expressed and purified, to a near homogeneity, the recombinant the CXXC domain of CXXC5 (CXXC-D) and the full-length CXXC5 protein (FL-CXXC5) using a bacterial expression system (Fig. 1a,c). To assess the DNA binding ability of the recombinant FL-CXXC5 in comparison with that of CXXC-D, we used isothermal titration calorimetry (ITC), which monitors heat changes caused by macromolecular, including DNA and protein, interactions. For the assay, 10 µM recombinant protein and 300 µM DNA were mixed in and subjected to ITC. The binding isotherms fitted to a one-site binding mode revealed that CXXC-D (Fig. 1b), as shown previously^7^, and FL-CXXC5 (Fig. 1d) binds effectively to a centrally located unmethylated ***CG*** dinucleotide bearing DNA (5′-GTGATAC***CG***GATCAGT-3′). Moreover, this binding was abolished when we used a DNA fragment containing identical sequences but with the methylated-***CG*** (***mCG***) dinucleotide (5′- GTGATAC***mCG***GATCAGT-3′) (Fig. 1e). The binding of FL-CXXC5 to the unmethylated ***CG*** containing DNA fragment is specific; because, a DNA fragment containing the central ***AT*** (5′-GTGATAC***AT***GATCAGT-3′), exhibited no binding (Fig. 1f). Furthermore, FL-CXXC5 also binds with similar affinities to DNA fragment with different sequences (5′- GAGAGAC***CG***GTCTCTC-3′) that surround the central ***CG*** (Fig. 1g) but not ***mCG*** (Fig. 1h) or ***AT*** (Fig. 1i) dinucleotide. These results suggest that FL-CXXC5 as its CXXC-D binds specifically to unmethylated CpG dinucleotide containing DNA.

**Figure 2:**
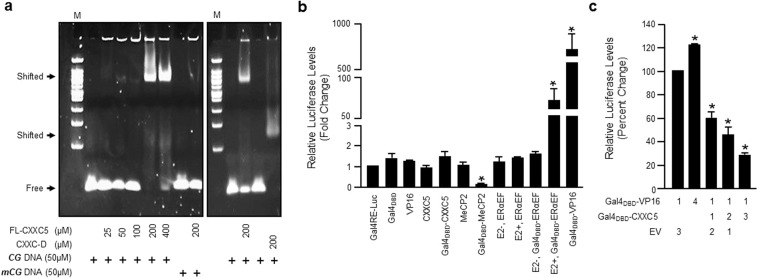
Electrophoretic mobility shift (EMSA) and reporter assay. (**a**) To assess the ability of the recombinant FL-CXXC5 or its CXXC domain (CXXC-D), 50 μM DNA with the central unmethylated (**CG**) or methylated (**mCG**) CpG dinucleotides was mixed with FL-CXXC5 at 1:0.5, 1:1, 1:2, 1:4 and 1:8 molar ratios or with CCCX-D at 1:4 molar ratios. Samples were run onto 5% native TBE gel, stained and visualized with UV spectrometry. “M” represents the molecular marker. “Free” denotes unbound DNA; “Shifted” indicates DNA bound protein. A representative experiment performed with two independent times is shown. **(b)** To assess the intrinsic transcription regulatory function of FL-CXXC5, pGal4-RE Luciferase Reporter vector (pGal4RE-Luc, 125 ng) containing tandem Gal4 response elements (Gal4-RE) juxtaposed to a simple TATA box promoter that drives the expression of the firefly Luciferase enzyme cDNA together with an expression vector (75 ng) bearing Gal4_DBD_, VP16, FL-CXXC5, WT-MeCP2 or ERα-EF domain cDNA or Gal4_DBD_-VP16, Gal4_DBD_-CXXC5, Gal4_DBD_-MeCP2 or Gal4_DBD_-ERαEF cDNA transfected into MCF7 cells. MCF7 cells grown in 10% CD-FBS for 48 h were transfected with expression vector bearing ERαEF cDNA or Gal4_DBD_-ERαEF and were treated without (%0.01 ethanol) or with 10^−8^ M E2 for 24 h. Transfection efficiency was monitored with the co-expression of a reporter plasmid bearing the *Renilla* Luciferase enzyme cDNA (0.5 ng). Results indicating relative firefly/*Renilla* luciferase activity determined using a dual luciferase assay kit are the mean ± S.E. of three independent experiments performed in duplicate. The asterisk denotes significant differences compared to responses observed with Gal4-RE, which is set to 1. **(c)** Cells were also transfected with pGal4RE-Luc together with the expression vector bearing none (empty vector, EV), Gal4_DBD_-VP16 and/or Gal4_DBD_-CXXC5 cDNA. In transfections, we used a total of 300 ng expression vector (1 denotes 75 ng), for which appropriate amounts of the parent expression vector (EV) were supplemented to equalize the total plasmid DNA amount. Results presented as relative firefly/*Renilla* luciferase levels indicate percent change and are the mean ± S.E. of three independent experiments performed in duplicate. The asterisk denotes significant differences from responses observed with Gal4_DBD_-VP16, which was set to 100%.

To further confirm these results, we also tested the ability of FL-CXXC5 and CXXC-D to bind specifically to an unmethylated CpG bearing DNA fragment with the central ***CG***, but not ***mCG***, sequence using electrophoretic mobility shift assay (EMSA) at 1:0.5, 1:1, 1:2, 1:4 and 1:8 molar ratio of DNA (50 μM) to FL-CXXC5 or 1:4 CXXC-D (Fig. 2a). EMSA, confirming ITC results, indicates that FL-CXXC5 as CXXC-D is an unmethylated CpG dinucleotide binder.

### **CXXC5 lacks a transcription activation/repression function**

Studies suggest that CXXC5 is a critical regulator/integrator of various signaling networks^4,10–12,17^. Functional studies also suggest that CXXC5 in a cell- and tissue-type dependent manner can act as a transcription activator^10,14,16^ or repressor^32^ to modulate gene expressions. These findings raise the possibility that CXXC5 has an intrinsic transcription regulatory function. To preliminary address this issue, we used a mammalian one-hybrid approach, a derivative of the mammalian two-hybrid system^33^, which exploits the modular nature of eukaryotic transcription factors that possess functionally distinct regions: DNA binding (DBD) and transcription activation domains. The binding of Gal4_DBD_, as the DNA binding module of a chimeric protein generated with genetic fusion, to Gal4 response elements (Gal4RE) preceding a simple TATA box promoter positions the protein to the promoter to drive the expression of the reporter enzyme: firefly *luciferase* from a Gal4RE-Luc reporter vector, as we described previously^34^. This approach, thereby, circumvents the need for the presence of transcription factor-specific responsive elements to drive reporter gene expressions in comparative analysis. We genetically fused FL-CXXC5 to the carboxyl-terminus of Gal4_DBD_ to generate the chimeric Gal4_DBD_-CXXC5 protein. We also used a chimeric transcription activator, Gal4_DBD_-ERαEF that bears the carboxyl-terminally located E/F domain of ERα^34^, which possesses a ligand (17β-estradiol, E2)-dependent transcription activation function. In addition, we utilized the Gal4_DBD_-VP16 fusion protein that bears the potent VP16 transcription activation domain. MeCP2 (methyl-CpG binding protein 2) was shown to act as a potent transcription repressor when fused to Gal4_DBD_^35^. Based on this finding, we also genetically fused MeCP2 to the Gal4_DBD_ to generate the Gal4_DBD_-MeCP2 chimeric protein. The proteins were then synthesized by transient transfections in E2-responsive MCF7 cells together with the Gal4RE-Luc reporter vector (Fig. 2B). If a protein has a transcription regulatory function, an augmentation/repression of reporter enzyme activity by a chimeric protein compared with levels induced by each protein alone is expected to occur. Results revealed that Gal4_DBD_-VP16 and Gal4_DBD_-ERαE/F, the latter which only in the presence of 10^–8^ M E2, effectively increased the reporter enzyme activity compared to that induced by Gal4_DBD_ alone. As shown previously^35^, Gal4_DBD_-MeCP2 robustly repressed the reporter enzyme levels. VP16, MeCP2 or ERαE/F, the latter which in the absence or presence of E2, alone have no effect on the enzyme activity. These results indicate that the positioning of a transcription regulator to a promoter independent of its DNA binding ability exposes the intrinsic transcription regulatory potential of the protein. In contrast, CXXC5 alone or as the Gal4_DBD_ fusion protein did not affect the levels of the reporter enzyme activity. To ensure that the fusion between Gal4_DBD_ and CXXC5 did not alter the ability of the chimeric protein to interact with promoter elements, we also carried out a promoter competition assay in MCF7 cells (Fig. 2c). Since Gal4_DBD_-VP16 potently enhances the reporter enzyme activity from the Gal4RE-promoter, a reduction in the extent of the reporter enzyme activity induced by Gal4_DBD_-VP16 through the competitive binding of Gal4_DBD_-CXXC5 to Gal4REs would indicate the ability of Gal4_DBD_-CXXC5 to bind to DNA. Indeed, the increasing concentrations of the expression vector bearing Gal4_DBD_-CXXC5, but not CXXC5 alone (data not shown), cDNA effectively decreased the reporter enzyme activity induced by Gal4_DBD_-VP16. Thus, these results indicate that CXXC5 lacks a transcription activation or repression function and suggest that CXXC5 may act as a nucleation factor for the establishment of a transcription state.

### The reduction of the intracellular CXXC5 level affects cell proliferation induced by E2

Our previous studies indicated that *CXXC5* is an E2-ERα responsive gene^3,4^. To assess the functional significance of CXXC5 through an augmented or a repressed level of CXXC5 synthesis in cell models derived from breast, or other tissues, adenocarcinomas, we used stable transfections with expression vectors bearing the CXXC5 cDNA or shRNA, the latter which was designed according to our previous study^4^. Unfortunately, however, the chronic over-expression or the repression of CXXC5 synthesis led to cell death independent of cell type or tissue-of-origin. This necessitated the use of an siRNA approach to examine the role of CXXC5 in MCF7 cells.

**Figure 3:**
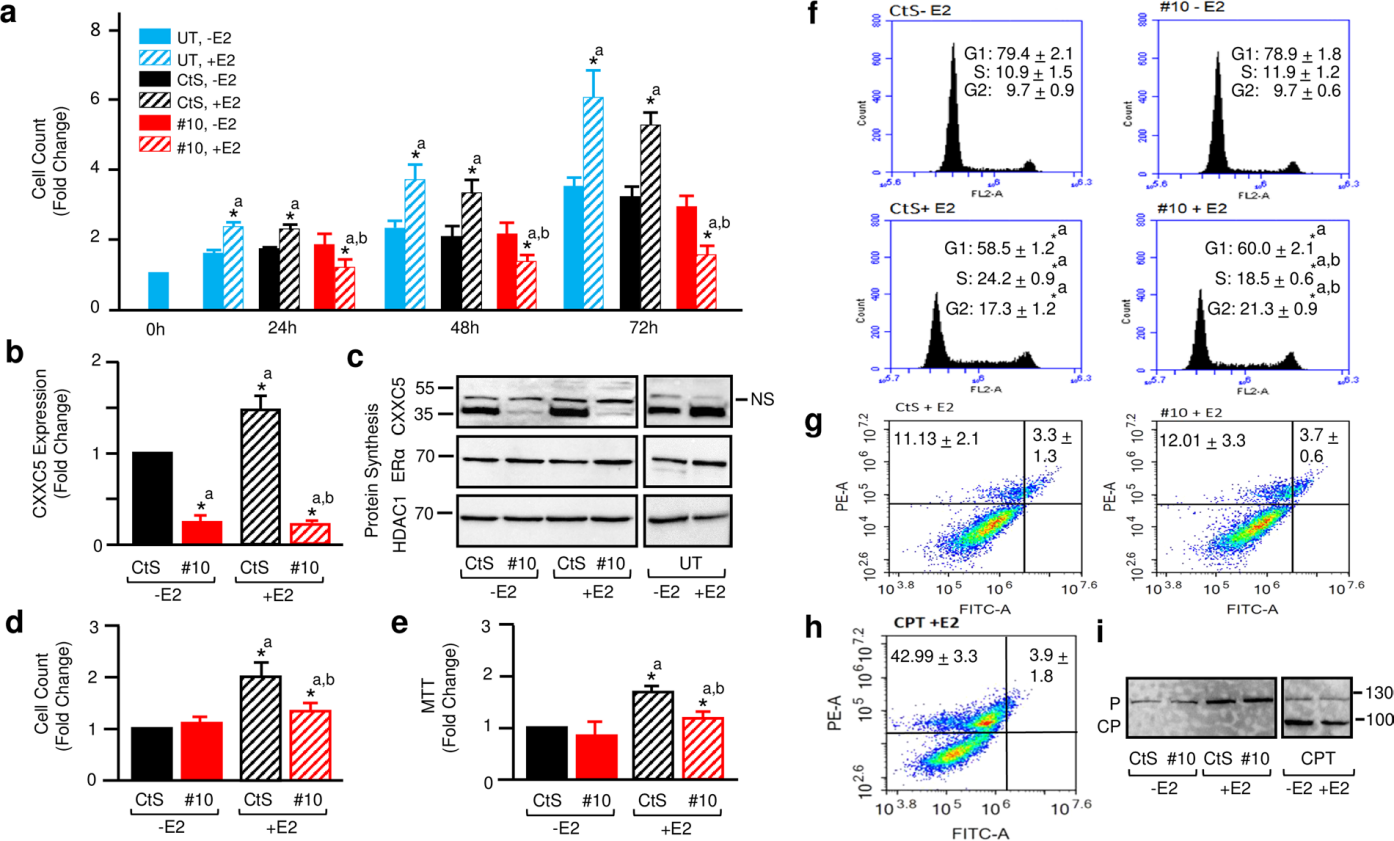
Effects of siRNAs specific to CXXC5 on cellular growth. MCF7 cells grown in 10% CD-FBS containing medium for 48 h were transiently transfected without (UT) or with 10 nM CtS or siRNA#10 in the absence (0.01% ethanol) or the presence of 10^−8^ M E2 up to 72 h. (**a**) Cells were subjected to cell counting using a hemocytometer. Results, as the mean ± S.E. of three independent determinations, depict fold change in cellular growth compared with those observed with cells at time 0 in the absence of E2, which is set to 1. In (**a–f**) the asterisk with a superscript “a” indicates significant difference from the corresponding ethanol-treated group (-E2); whereas superscript “b” depicts a significant difference from CtS transfected cells in the presence of E2. (**b**) The cDNA library generated from total RNA (50 ng) isolated from transfected cells for 48 h was subjected to qPCR using a primer set specific to CXXC5. (**c**) Nuclear extracts from untransfected (UT) or transfected cells were subjected to WB using an antibody for CXXC5, ERα or HDAC1. NS denotes non-specific protein; Molecular mass in KDa is indicated. Transfected cells were subjected to cell counting (**d**) and MTT assay (**e**). In (**b**,**d**,**e)**, results depicting the mean ± S.E. of three independent determinations indicate fold change in mRNA levels (**b**) or cell numbers (**d**,**e**) compared with CtS in the absence of E2, which is set to 1. Transfected MCF7 cells were subjected to flow cytometry (**f**) with results depicting the percent of cells in the G1, G2 and S phases, or to Annexin V assay followed by flow cytometry (**g**). In (**f**,**g**), results are the mean ± SEM of three independent experiments. (**h & i**) Untransfected (UT) or transfected cells were also subjected to the cell death inducer camptothecin (2 nM) for 24 h without (−E2) or with E2 (E2). Cells were then subjected to Annexin V assay (**h**) with results as the mean ± S.E. of three independent experiments depicting percent change in apoptotic (upper right quadrant) and death (upper left quadrant) cells, or to WB (**i**) using a PARP1-specific antibody. P and CP denote the uncut and cut PARP1, respectively.

We, therefore, carried out studies with siRNAs to assess CXXC5 functions in MCF7 cells treated without or with E2. In our previous study^4^, we had evaluated four different siRNAs that target CXXC5 transcripts (FlexitubeGene Solution, Qiagen siRNA #2, 7, 9 and 10) as well as a scrambled siRNA control (CtS) to alter levels of CXXC5 transcript and protein. Of the siRNAs, siRNA#10 and siRNA#2 (for siRNA sequences: Supplementary Table S1) effectively repressed CXXC5 transcript levels and protein synthesis in MCF7 cells in comparison with CtS, which had no effect on CXXC5 transcript or protein levels compared to untransfected (UT) cells^4^. Based on these findings, we assessed the effect of siRNA#10 initially on cellular growth. MCF7 cells grown in 10% CD-FBS containing medium for 48 h to reduce endogenous E2 levels, were transiently transfected without or with CtS or siRNA#10 in the absence (-E2; ethanol as vehicle control, 0.01%) or the presence of E2 (E2; 10^–8^ M) up to 72 hours (Fig. 3a). We found by cell counting that E2 treatment of UT cells, as expected, increased the cellular proliferation at every time point tested. CtS did not adversely affect cell number compared to that of UT cells in the absence or presence of E2. On the other hand, siRNA#10 effectively prevented specifically E2-mediated cellular growth at time points tested. Similar results were also observed with siRNA#2 (Supplementary Fig. S2a).

Based on these initial results, MCF7 cells maintained in 10% CD-FBS for 48 h were transiently transfected with CtS or siRNA#10 in the absence or presence of 10^–8^ M E2 for 48 h. E2, as we showed previously^4^, augmented the expression and protein synthesis of CXXC5 in cells transfected with CtS. siRNA#10, on the other hand, effectively reduced CXXC5 at both transcript (Fig. 3b) and protein (Fig. 3c) levels in the absence or presence of E2. siRNA#10 did not adversely affect the protein level of ERα, nor that of HDAC1 used as a loading control, that could account for cellular events mediated by E2 in transfected cells (Fig. 3c).

Confirming our initial results (Fig. 3a), we also observed that siRNA#10 reduced cellular proliferation only in the presence of E2 assessed by cell counting (Fig. 3d) or MTT assay (Fig. 3e). The E2-induced proliferation of MCF7 cells is mediated by ERα, as ICI (Imperial Chemical Industries 182,780), an ER antagonist, effectively prevented the growth of the cells transfected with CtS or siRNA#10 (Supplementary Fig. 2b). siRNA#10 also repressed the E2-mediated growth of ERα-synthesizing T47D cells derived from a breast ductal carcinoma (Supplementary Fig. S2c). These findings indicate that CXXC5 is involved in the cellular proliferation mediated by the E2-ERα signaling.

### The reduction of the intracellular CXXC5 level alters cell cycle progression

To examine whether or not the reduction of E2-mediated cellular proliferation by siRNA#10 involves alterations in cell cycle phases, we transiently transfected MCF7 cells, grown in 10% CD-FBS containing medium for 48 h, with CtS or siRNA#10 in the absence (-E2) or the presence of E2 (E2; 10^−8^ M) for 48 h (Fig. 3f). E2 increased the cell population in the S and G2 phases with a concomitant decrease in the G1 phase in cells transfected with CtS. siRNA#10 reduced the E2-mediated augmentation of the cell population in the S phase and increased the population in the G2 phase without an effect on the cell number in the G1 phase. These results suggest that CXXC5 contributes to events in the E2-driven S phase and in the G2/G1 transition.

To assess whether or not the repression of cellular proliferation by siRNA#10 includes also cell death, MCF7 cells grown in 10% CD-FBS containing medium for 48 h were transiently transfected with CtS or siRNA#10 in the absence or presence of E2 (E2; 10^−8^ M) for 48 h. Cells were then subjected to an Annexin V assay followed by cytometry as well as WB for the cleavage of poly (ADP-ribose) polymerase-1 (PARP1) as a marker for cell death. Results revealed that siRNA#10 in the absence or presence of E2 (shown in the presence of E2) had no effect on cellular death compared to cells transfected with CtS nor did it affect the cleavage of PARP1, the level of which was increased in the presence of E2 (Fig. 3g,i). In contrast, camptothecin (CPT), a DNA topoisomerase inhibitor, effectively increased death cell populations (shown in the presence of E2) (Fig. 3h) and cleaved PARP1 (CP) in the absence or presence of E2 (Fig. 3i). These results collectively suggest that CXXC5 as an unmethylated CpG dinucleotide binder contributes to cellular proliferation mediated by E2-ERα signaling through events occurring primarily in the S phase of the cell cycle.

### CXXC5 alone and together with E2-ERα is involved in the regulation of gene expressions

**Figure 4:**
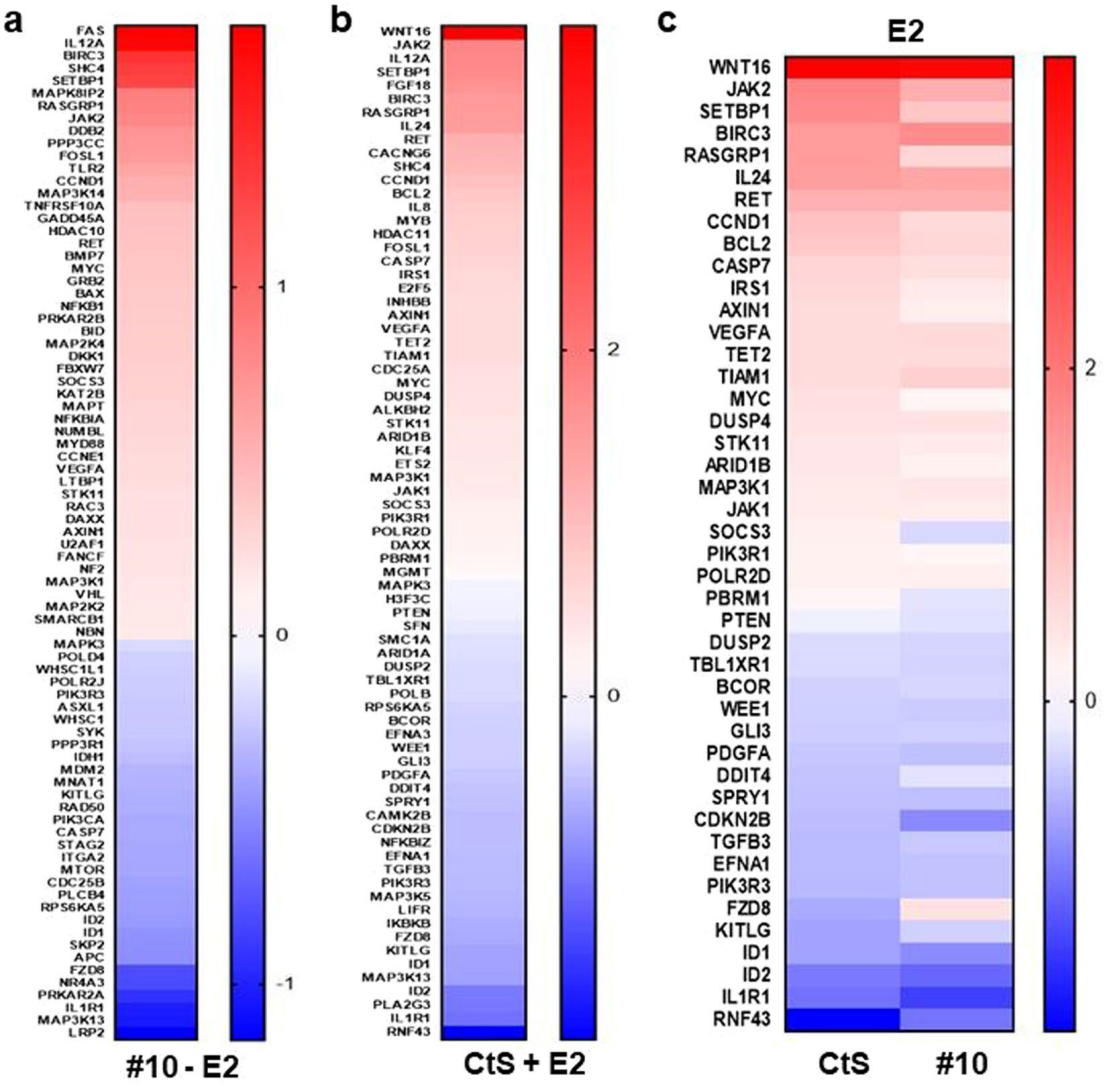
Analysis of Gene Expression with the nCounter PanCancer Pathway Panel. MCF7 cells grown in 12-well tissue culture plates (9 × 10^4^ cells/well) in 10% CD-FBS medium for 48 h were transiently transfected using 10 nM of CtS or siRNA#10, generating four treatment groups: Group (1) CtS and −E2; (2) #10 and −E2; (3) CtS and +E2; (4) #10 and +E2. Forty-eight hours after transfection, cells were treated without (ethanol, 0.01%) as vehicle control or with 10^−8^ M of E2 for 3 hours. Total RNA (50 ng) was subjected to the nCounter PanCancer Pathway Panel gene expression analysis with nCounter Digital Analyzer. Quality control, data normalization and differential expression analyses were carried out using Nanostring nSolver 3.0 Analysis software together with its Advanced Analysis plug-in. Each treatment is depicted as heat maps with increasing (red) and decreasing levels (blue) and are the mean of three independent experiments in the log2 scale. **(a)** To assess differentially expressed genes mediated by CXXC5 in the absence of E2 (#10 −E2), nanostring results obtained from cells transfected with siRNA#10 in the absence of E2 (Group 2) was normalized to those from cells transfected with CtS in the absence of E2 (Group 1). **(b)** The effects of E2 on gene expression (CtS and +E2) were assessed by normalizing nanostring results obtained from cells transfected with CtS in the presence of E2 (Group 3) to those from cells transfected with CtS in the absence of E2 (Group 1). **(c)** To reveal the impact of CXXC5 on gene expressions in response to E2, we compared differentially expressed genes observed in the presence (CtS and +E2) and the absence (#10 and +E2) of CXXC5, the latter which was obtained by normalizing nanostring results obtained from cells transfected with siRNA#10 in the presence of E2 (Group 4) to those from cells transfected with siRNA#10 in the absence of E2 (Group 2).

The contribution of CXXC5 to the E2-mediated cellular proliferation together with our observation that CXXC5 is an unmethylated CpG binder raises the possibility that CXXC5 alone or together with E2-ERα alter gene expressions. To test this prediction, we carried out a multiplex gene expression analysis using the PanCancer Pathway Panel (nCounter, Nanostring) that contains 770 genes from cancer-associated canonical pathways involved in cell cycle regulation, apoptosis, DNA damage control, transcriptional regulation, and chromatin modification. This approach provides direct measurements of cellular levels of mRNA transcripts^36,37^. MCF7 cells grown in 10% CD-FBS containing medium for 48 h were transiently transfected with CtS or siRNA#10 in the absence of E2. Forty-eight hours after transfection, cells were treated without (-E2, 0.01% ethanol) or with 10^−8^ M E2 (+E2) for 3 h, which is a time period critical for the assessment of changes in the expression of primary (protein synthesis independent) E2 target genes^38,39^. This treatment regimen generated four groups: Group 1) CtS and -E2; 2) #10 and -E2; 3) CtS and +E2; 4) #10 and +E2. Total RNA from each group was processed for and subjected to the Panel profiler. Following background correction, we assessed gene expressions mediated by CXXC5 in the absence of E2, Group 2 by normalizing to Group 1 (Fig. 4a & Table S2). Results revealed that CXXC5 in the absence of E2 modulates the expression of a number of genes including *APC, BAX, BID, CCNE1, BMP7, DKK1, GADD45A, GRB2, HDAC10, ITGA2, KAT2B, MTOR, NF2, NFKB1, NFKBIA, POLD4, POLR2J, RAD50, SKP2 and SMARCB1*, whose encoded proteins are involved in various intracellular events ranging from signal transduction to chromatin modeling. We also normalized Group 3 to Group 1 to reveal genes differentially regulated by E2 when CXXC5 is present (Fig. 4b & Table S2, Column 2). This group of genes includes *AXIN1, BCL2, CCND1, CDKN2B, DUSP4, ETS2, ID1, ID2, IL24, IRS1, MAP3K5, MGMT, MYC, NFKBIZ, PDGFA, PTEN, TET2, and TGFB3*, which were shown to be regulated by E2^3,39–50^. The normalization of Group 4 to Group 2, on the other hand, indicates genes mediated in response to E2 in the absence of CXXC5 (Table S2), which, in addition to E2-regulated genes, also include the appearance of genes encoding the components of Interleukin (*IL1RAP*), Growth Factors (*ERBB2, LTBP1, TGFB2, VEGFC*), Wnt (*FZD7, WNT7B*), Notch (*NOTCH3, JAG2*) and Hedgehog (*PTCH1*) signaling pathways. To further reveal the impact of CXXC5 on gene expressions in response to E2, we then compared E2-mediated gene expressions observed in the presence (Group3/Group1) and the absence (Group4/Group2) of CXXC5. Results revealed that CXXC5 participates in the expression of a set of E2-responsive genes antagonistically or additively (Fig. 4c). CXXC5, for example, antagonistically modulated the expression of *FZD8*, *PBRM1, SOC3* in the presence of E2. CXXC5, on the other hand, additively modulated (augmented or attenuated) the expression of *AXIN1, CCND1*, *CDKN2B, IL24, IRS1, MYC, PTEN, RET, RINF43* or *TGFB* in response to E2. These results collectively indicate that the integrated effects of CXXC5 and E2-signaling define the state and the level of target gene transcriptions and suggest that CXXC5 is a critical regulatory component for the fine-tuning of E2-mediated gene expressions, thereby of cellular proliferation.

### *ERα* expression is correlated with the expression of *CXXC5*, whose overexpression is associated with poor prognosis in ERα-positive (ER**+**) breast cancer

**Figure 5:**
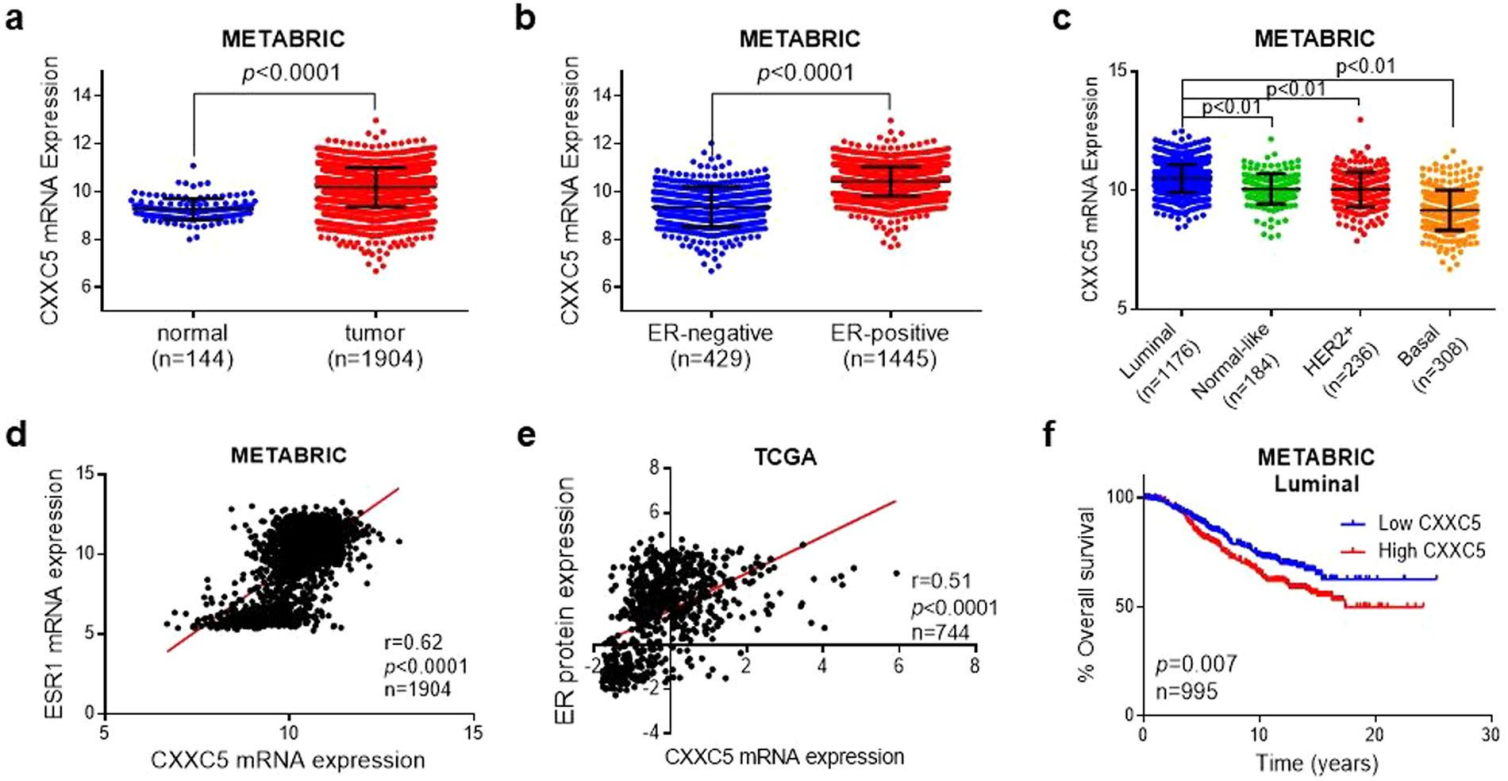
Clinicopathological relevance of *CXXC5* expressions in breast cancer patients. (**a**) *CXXC5* expression in breast cancer (BC) as compared to normal breast tissue from METABRIC. **(b)** *CXXC5* expression in ER-negative versus ER-positive breast cancer patients from METABRIC. **(c)** *CXXC5* expression in different molecular subtypes of tumors of breast cancer patients from METABRIC. **(d)** Pearson correlation analysis between mRNA expressions of CXXC5 and ESR1 (ERα) in patients from METABRIC. **(e)** Pearson correlation analysis between mRNA expression of *CXXC5* and ERα protein level in breast cancer patients from TCGA. **(f)** Kaplan Meier survival analysis of basal breast cancer patients based on *CXXC5* expression separated from the median.

Since *CXXC5* is an E2-ERα responsive gene and the CXXC5 protein appears to be critical for E2-mediated cellular events in MCF7 cells modeling ER-positive breast cancer, we wanted to assess whether there is a correlation between the *CXXC5* and *ESR1* (ERα) expressions and whether the *CXXC5* expression has a prognostic value in ER+ breast cancer patients using two large, publically available breast cancer patient datasets, METABRIC (Molecular Taxonomy of Breast Cancer International Consortium) and TCGA (The Cancer Genome Atlas). We found that the mean *CXXC5* expression is significantly higher in breast cancer than that observed with normal breast tissue (Fig. 5a). Previous studies have indicated that the overexpression of *CXXC5* is associated with poor prognosis in ERα-positive (ER+) breast cancer^25,51^. Consistent with this, we found that the mean of *CXXC5* expression is higher in ERα+ luminal breast tumors compared to that observed with normal-like and ERα-negative breast cancer subtypes, including HER2+ and Basal-like tumors (Fig. 5b,c). Moreover, mRNA levels of *CXXC5* positively correlate with both mRNA and protein levels of ERα in breast cancer patients (Fig. 5d,e). Importantly, higher levels of the mean *CXXC5* expression predict a poor overall survival only in luminal patients while the expression levels of *CXXC5* are not prognostic in HER2+ or Basal-like subtypes (Fig. 5f, Supplementary Fig. S5), further supporting its clinical relevance to ER+ breast cancer. These results suggest that there is a correlation between *CXXC5* and *ESR1* expressions in ER+ breast cancer and that a deregulated expression of *CXXC5* could contribute to the initiation and/or progression of ERα+ breast cancer.

## Discussion

In assessing the functions of CXXC5 *in vitro* and *in cellula* we show here that CXXC5 is an unmethylated CpG dinucleotide binding protein and contributes to gene expressions and, consequently, to the modulation of cellular proliferation driven by the E2-ERα signaling.

In vertebrates, DNA methylation by DNA methyltransferase enzymes (DNMTs) is one of the mechanisms of gene silencing and it mostly occurs in CpG dinucleotides of the genome. Methylation of cytosine residues results in the recruitment of methyl-CpG-binding proteins (MBPs) that act as transcription repressors^52^. Although the majority of CpGs in mammalian genomic DNA is methylated^53^, about 70% of human gene promoters are associated with unmethylated DNA sequences called CpG islands (CGIs)^54–56^. CGIs are rich in C and G nucleotides and are defined by a high density of CpG dinucleotides. CGIs are often found in the promoter regions of genes that have characteristic transcription-associated chromatin organizations and are mostly hypomethylated. Recent structural studies indicated that the ZF-CXXC domains of the ZF-CXXC family proteins recognize and bind to unmodified CpG dinucleotides with varying affinities and specifies allowing to be grouped in distinct classes^7^. For example, MLL2 (Mixed-lineage leukemia 1 & 2); KDM2A and KDM2B (Lysine (K)-specific Demethylase 2A & 2B); FBXL19 (F-box and leucine rich repeat protein 19); MBD1 (Methyl-CpG-binding domain protein 1) as the Class II ZF-CXXC family proteins specifically recognize unmethylated CpG-containing DNA. The ZF-CXXC domains of TET1 and TET3 (Tet methylcytosine Dioxygenase 1 & 3), CXXC4 (CXXC-type zinc finger protein 4), CXXC5 bind to cytosine containing DNA with a preference for CpG over CpH (H any nucleotide other than G). On the other hand, CFP (CXXC finger protein 1) as the only member of Class I binds to CpGpGp sequence with high affinity. Whereas, the CXXC domain of DNMT1 (DNA (cytosine-5)-methyltransferase 1) as the Class IV protein displays a weak or no binding to DNA^7^. Although the CXXC domain of CXXC5 interacts with CpH as well as methylated CpG containing DNA, the binding affinity of the CXXC domain to these sequences is considerably lower (10–40 fold) than those observed with sequences containing the unmethylated CpG dinucleotide^7^. On the other hand, we observed here that the full-length CXXC5 binds specifically to unmethylated CpG dinucleotide containing DNA. As suggested for various proteins^31^, it is likely that intramolecular allosteric interactions between the disorder amino-terminus and the carboxyl-terminally located CXXC domain, or the presence of an affinity modulatory sub-region within the amino-terminus, provide selectivity to apoCXXC5 to interact primarily with unmethylated CpG dinucleotides. Dynamic conformational fluctuations in CXXC5 mediated by protein-protein interactions and/or post-translational modifications in a cellular environment could also be critical for the protein to recognize and bind to a permutation of unmodified or modified cytosine-centered DNA sequences in a signal- and cell type-specific manner.

Our results also indicate that the E2-responsive gene product CXXC5 is a critical contributor to the expression of genes mediated by the E2-ERα signaling. The expression of the primary E2 responsive genes by E2-ERα encompasses proteins involved in the metabolism of nucleic acid/proteins as well as transcription factors, membrane signaling cascade and receptor proteins. These proteins, in turn, participate in the regulation of late gene expressions responsible for the modulation of DNA replication, recombination, and repair, cell cycle, and division, consequently in the initiation of E2-mediated cellular proliferation^38,57–59^. Although limited in the gene number, our multiplex gene expression analysis using the PanCancer Pathway Panel revealed that the suppression of CXXC5 levels in cells without an active E2 signaling, i.e. cells transfected with siRNA#10 in the absence of E2, results in the regulation of a number of genes, including *CCND1, CCNE1, CDC25B, MYC* critical for cellular proliferation. However, the modulatory effect of CXXC5 on gene expressions was not reflected in the proliferation, as we observed no change in cellular growth compared to cells transfected with CtS in the absence of E2. On the other hand, E2 treatment of cells transfected with CtS effectively increased cellular proliferation through the expression of a set of genes including *CDC25A*, *MYB*, and *WEE1* specifically regulated by E2 as well as *CCND1* and *MYC* regulated by both E2 and CXXC5. In contrast, the diminished CXXC5 levels in cells resulted in an effective attenuation of E2-driven cellular proliferation. In addition to genes regulated independently as well as commonly by E2 and CXXC5 critical for cellular proliferation, the appearance of a set of genes modulated by E2 as a result of the diminished synthesis of CXXC5 that includes *CDK6*, which encodes a cyclin-dependent kinase critical for the initiation and transition of cell cycle phases and *ATR*, which encodes a serine/threonine-protein kinase involved in checkpoint control, could underlie the repression of E2-driven cellular proliferation. This implies that CXXC5 contributes to the regulation of not only known but also latent E2 responsive gene expressions critical for cellular proliferation.

Although it is clear that CXXC5 is involved in gene expressions, the underlying mechanism by which CXXC5 mediates transcription remains conjectural. Our findings that CXXC5 lacks a transcription regulatory function but is an unmethylated CpG dinucleotide binder suggest that upon binding to DNA, CXXC5 could act as a nucleation factor to establish a transcription state permissive or restrictive for transcription by preventing DNA methylation and/or recruiting epigenetic and/or transcription factors. In keeping with this prediction, our ongoing studies suggest that CXXC5 indeed interacts with various transcription factors as well as DNA and histone modifiers. This is also consistent with findings that the genetic ablation of *CXXC5* in a mouse model results in epigenetic alterations, including decreased levels of promoter CpG methylation as well as histone modifications, at the Interferon regulatory factor 7 gene (*IRF7*) locus in plasmacytoid dendritic cells^24^. Likewise, the loss of CXXC5 was recently shown to alter the methylation state of embryonic stem cells contributing to altered gene expressions^19^. Furthermore, CXXC5 was reported to inhibit the expression of the CD40 ligand gene (*CD40L*) by interacting with histone-lysine N-methyltransferase SUV39H1 at the gene promoter^60^. Extending these, our observations based on a publicly available methylation profiling data set generated by the use of genome tiling arrays carried out with DNA from MCF7 cells in the absence or the presence of E2 (GSE132513) suggest that the methylation state of some of the estrogen-responsive genes and their expressions could correlate with the presence or absence of CXXC5 (Supplementary Fig. S5). We observed that *MGMT*, *DAXX*, *CACNG6*, *HDAC11* and *SHC4* expressions, which are upregulated by E2 only in the presence of CXXC5 but remain unchanged when CXXC5 is knockdown (Fig. 4, and Supplementary Table 2), tend to be hypomethylated upon E2 stimulation in MCF-7 cells. Of these, the hypomethylated regions of *CACNG6* or *SHC4* are located at the promoter. On the other hand, the expression of *NFKBIZ, IKBKB, CAKM2B, MAP3K13, RPS6KA5* or *POLB* which is downregulated by E2 only in the presence of CXXC5 but remains unaltered when CXXC5 is knockdown (Fig. 4, and Supplementary Table 2), is likely to be hypermethylated at the promoter regions. While its importance remains to be explored, we also observed *in vitro* that CXXC5 binds to hemimethylated CG containing DNA fragment (Supplementary Fig. S6). This raises an intriguing possibility that CXXC5 could also be involved in the maintenance of replication, as suggested for DNMTs^61,62^ as well as monoallelic expression patterns of imprinted genes, which are lost in human cancer^63^.

Interestingly, some latent E2-responsive genes encode protein components of signal transduction pathways mediated by the cytokine, growth factor and frizzled family of proteins, which were previously shown to drive the expression of *CXXC5*^9–14^. This suggests that the integrated effects of various signaling pathways at both basal- and induced-state determine the magnitude and direction of the *CXXC5* expression. Since cell type-specific gene expression programs are controlled by the repertoire of lineage-specific transcription regulators responding to distinct stimuli, altered levels of the *CXXC5* expression would also lead to diverse cellular responses ranging from proliferation to differentiation and death, as reported previously^8–10,12,15–19,32^, in a cell- and tissue-specific manner, contributing to the physiology and pathophysiology of various tissue functions.

We, as others^25,51^, find that high levels of *CXXC5* expression predict poor overall survival, particularly in ERα-overexpressing luminal breast cancer patients. This is consistent with the prediction that the overexpression of *CXXC5* represents an independent molecular marker for the unfavorable prognosis in ER+ breast tumors^25,51^. Moreover, we find here that the *CXXC5* expression also correlates with the mRNA and protein levels of ERα. Since *CXXC5* is an E2-ERα responsive gene, CXXC5 could indeed participate in the initiation and progression of breast cancer driven by a de-regulated E2-ERα signaling. This, in turn, implies that in addition to being a prognostic factor, CXXC5 could be an important therapeutic target for the disease as well.

## Methods

### Cell culture and biochemicals

The growth and maintenance of E2 responsive and ERα-synthesizing MCF7 and T47D cells were described previously^4,64^. In all experiments, media were changed every third day when appropriate.

Restriction and DNA modifying enzymes were obtained from New England Bio-Labs (Beverly, MA, USA) or Thermo-Fischer Sci. (Waltham, MA, USA). 17β-estradiol (E2) was purchased from Sigma-Aldrich (St. Louis, MO, USA). The complete antagonist of estrogen receptor, Imperial Chemical Industries 182780, (ICI) was obtained from Tocris Biosciences (Ellisville, IL, USA). The antibody for Poly(ADP-ribose) polymerase 1 (PARP1; 9542) was obtained from Cell Signaling Technology (Beverly, MA, USA). The antibodies for CXXC5 (ab106533) and for HDAC1 (ab19845) were purchased from Abcam Inc. (Cambridge, MA, USA). Secondary antibodies conjugated with horseradish peroxidase were obtained from Santa Cruz Biotech (Santa Cruz, CA, USA). siRNAs (FlexiTube siRNA) for CXXC5 were purchased from Qiagen Inc. (Düsseldorf, Germany). Camptothecin (13637) was purchased from Cell Signaling Technology.

### Cloning of cDNA for the full-length CXXC5 or the Zinc Finger CXXC domain into the pET28a-MHL vector for bacterial expression

The expression vector bearing the human CXXC5 cDNA was described previously^4^. The sequences encoding the full-length CXXC5 (322 aa) or the CXXC DNA binding domain (250–309 aa) generated by PCR using the CXXC5 cDNA as the DNA template were inserted into the pET28a-MHL vector (GenBank accession EF456735) with appropriate restriction enzyme sites. The vector contains sequences encoding the 6xHis tag at the 5′ end of the inserted cDNA followed by sequences for the cleavage with Tobacco Etch Virus (TEV) protease for the initial protein purification. TEV protease was produced at the Structural Genomics Consortium (University of Toronto, Toronto, CA). The vector also contains the kanamycin resistance gene for antibiotic selection.

### Protein expression and purification

The recombinant full-length CXXC5 or the CXXC domain protein was overexpressed in *Escherichia coli* BL21(DE3)-V2R-pRARE2 strain (Structural Genomics Consortium; Toronto, CA) in terrific broth (Sigma-Aldrich, MO, USA) supplemented with 0.8% glycerol, 50 µg/ml kanamycin, 17 µg/ml chloramphenicol, 50 µM ZnCl_2_ and 800 µl antifoam-204 (Sigma-Aldrich) in a 2-liter flask and grown at 37 °C with shaking at 200 rpm. When OD_600_ reached 1.6, the temperature was reduced to 16 °C and 250 µM IPTG was added into the culture to induce protein synthesis. Cultures were aerated overnight at 16 °C with shaking at 200 rpm. Cells were collected by centrifugation at 4 °C, lysed in a lysis buffer (50 mM Tris pH 8.0, 500 mM NaCl, 1 mM PMSF, 3 mM β-mercaptoethanol, 50 µM ZnCl_2_ and benzonase) and homogenized using an Ultra-Turrax T8 homogenizer at a maximal setting for 60 seconds. The slurry was then sonicated briefly and was centrifuged at 15,000 rpm for 1 h at 4 °C to clear lysate. The cleared supernatant subsequently mixed with 10 ml of equilibrated His-Select resin (Sigma-Aldrich) and incubated for 30 min at 4 °C with continuous mixing. The mixture was then loaded onto a column and washed with 300 ml wash buffer (50 mM Tris pH: 8.0, 2 M NaCl, 13 mM imidazole pH: 8.0, 3 mM β-mercaptoethanol, 50 µM ZnCl_2_). Proteins were eluted from the resin by 20 ml of elution buffer (50 mM Tris pH: 8.0, 300 mM NaCl, 250 mM imidazole pH: 8.0, and 1 mM TCEP). To remove the high concentration of imidazole from the protein mixture, the mixture was dialyzed against 50 mM Tris pH 8.0, 150 mM NaCl and 1 mM TCEP at 4 °C overnight. During the dialysis procedure, TEV enzyme (1 µg TEV for 10 µg recombinant protein) was added directly into the protein mixture to remove the 6xHis tag from proteins. Protein samples were subsequently subjected to a HiTrap SP Fast Flow (GE Healthcare, Sigma-Aldrich) ion-exchange chromatography using buffers with gradient salt concentrations (Buffer A: 50 mM Tris pH 8.0, 20 mM NaCl, 1 mM TCEP, Buffer B: 50 mM Tris pH 8.0, 1 M NaCl; 1 mM TCEP). Fractions corresponding to protein peaks were loaded on SDS-PAGE (4–20% gradient) and stained by InstantBlue coomassie dye (Expedeon, San Diego, CA, USA) to ensure the protein purification procedure. Protein fractions were then collected and subjected to a Superdex 200 (10/300) gel filtration (size exclusion) column (GE Healthcare Bio-Sci., Pittsburgh, PA, USA) followed by a HiTrap Heparin HP (GE Healthcare) column for further purification. Eluted proteins in an elution buffer (20 mM Tris pH 7.5, 150 mM NaCl and 1 mM TCEP) were subjected to SDS-PAGE (4–20% gradient) and stained by the InstantBlue coomassie dye to ensure the purification procedure. The protein fractions that contained the full-length CXXC5 or the CXXC domain protein were collected and concentrated using 10K-MW (for the FL-CXXC5) and 3K-MW (for the CXXC-D) cut-off concentrators (Thermo-Fisher) for functional assays.

### Isothermal titration calorimetry (ITC) binding assay

The abilities of the purified proteins to interact with DNA were assesses with ITC. Oligonucleotides containing a central CpG dinucleotide without or with methyl-cytosine residue or an AT dinucleotide as single-stranded DNA were purchased from Integrated DNA Technologies (IDT). Each pair of single-strand DNAs, dissolved in buffer 20 mM Tris pH 7.5 and 150 mM NaCl, was mixed in a 1:1 molar ratio, heated to 95 °C for 5 min and cooled to room temperature to obtain a double-stranded DNA. The full-length CXXC5 or the CXXC domain protein at the concentration of 10 µM and of 300 µM DNA were used in the ITC experiment using a VP-ITC (Malvern Panalytical, Malvern, UK) at 25 °C. A total of 25 injections was performed with 180-sec intervals using the reference power of 15 μcal/sec. Binding isotherms were plotted and analyzed using Origin 7.0 software (MicroCal Inc.). ITC measurements were fit to a one-site binding model.

### Electrophoretic mobility shift assay (EMSA)

We also assessed the abilities of the recombinant FL-CXXC5 or the CXXC-D protein to bind to DNA with EMSA. EMSA was carried out as described previously^4,65^. As detailed for ITC experiments, the double-stranded DNA without or with methyl-cytosine residue, 50 µM, was mixed with the full-length CXXC5 or the CXXC domain protein in increasing molar ratios and incubated for 30 min on ice. The samples were then loaded onto 5% TBE ready gels (Bio-Rad Laboratories, CA, USA) in 0.5X TBE running buffer. The gel was stained by the SybrGold dye (Thermo-Fisher) for visualization.

### Transfections

For siRNA transfection, cells (in 96-well tissue culture plates for cellular growth; in 12-well tissue culture plates for RNA extraction or 6-well tissue culture plates for WB and phenotypic assays) were cultured in charcoal-dextran treated fetal bovine serum-containing (CD-FBS) phenol red-free medium for 48 h to reduce/ablate steroid hormone levels. Cells then were transiently transfected with the HiPerfect transfection reagent (Qiagen) using 10 nM of a scrambled siRNA (AllStar, CtS) or of an siRNA specific for CXXC5 (FlexiTube GeneSolution, Qiagen) and treated in the absence (ethanol, 0.01%) or the presence of 10^−8^ M E2 and/or 10^−6^ M ICI, which is a complete ER antagonist, as we described previously^4^ for the duration of an experiment defined in the result section. The amount of siRNA we selected was based on our initial studies, with which we observed that 10 nM was the maximal amount of the control siRNA (CtS) without inducing phenotypic changes in transfected MCF7 cells up to 96 h.

### Luciferase assay

To assess whether or not CXXC5 has a transcription activation or repression activity, we used luciferase assays. pGal4-RE Luciferase Reporter vector (pGal4-RE-Luc) contains a tandem Gal4 response elements (Gal4-RE) juxtaposed to a simple TATA box promoter that drives the expression of the *Firefly* Luciferase enzyme cDNA as the reporter enzyme (Promega Corp., Madison, WI, USA), as we described previously^34^. The expression vector (pM) bearing sequences encoding the GAL4 DNA Binding Domain, DBD, (Gal4_DBD_, amino acids 1–147), and a nuclear localization signal was used as the template to generate fusion proteins. The sequences encoding the FL-CXXC5, full-length MeCP2 (amino acids 1–486) or the multifunctional carboxyl-terminus of ERα-EF domain containing the ligand-dependent transaction activity (amino acids 301–595) were inserted into the 3′ end of the sequences encoding the GAL4_DBD_ with appropriate restriction enzyme sites. We also used an expression vector bearing only the FL-CXXC5, FL-MeCP2 or ERαEF cDNA as control. Expression vectors (75 ng/transfection) were transfected into MCF7 cells together with the pGal4-RE-Luc reporter plasmid (125 ng/transfection) as described previously^4^. Transfection efficiency was monitored with the co-expression of a reporter plasmid that drives the expression of the *Renilla* Luciferase enzyme cDNA (0.5 ng). In transfections using the expression vector bearing ERαEF or Gal4_DBD_-ERαEF cDNA, cells were treated without (ethanol, 0.01%) or with 10^−8^ M E2. Twenty-four hours after transfections, cellular extracts were assayed for the change in luciferase enzyme activities using a Dual-Luciferase Assay kit (Promega Corp.), as described previously^4^.

### PCR and RT-qPCR

Isolated total RNA from cells was used for the cDNA synthesis (The RevertAid First Strand cDNA Synthesis Kit, Thermo-Fisher). The SYBR Green Mastermix (BioRad, Hercules, CA, USA) and gene-specific primers (Supplementary Table S1) were used for RT-qPCR reactions on BioRad Connect Real-Time PCR, as we described previously^4^. For the normalization of results, we used the expression of *RPLP0* (60 S acidic ribosomal protein P0), a reference gene for the normalization of E2-regulated genes in breast carcinomas^66^. The relative quantitation of gene expressions was assessed with the comparative 2^-ΔΔC^_T_ method^67^. During the RT-qPCR experiments, MIQE Guidelines were followed^68^.

### Western blotting (WB)

WB was performed as described previously^4^. In brief, cells grown in six-well tissue culture plates in medium supplemented with 10% CD-FBS to reduce the endogenous E2 concentration for 48 h were transfected with an siRNA specific for CXXC5 or a non-target control siRNA (AllStar, CtS) in the absence (EtOH, 0.01%) or the presence of E2 (10^−8^ M) for 48 h. At the termination, cells were collected and the nuclear content was isolated using NE-PER protein extraction kit (Thermo Fisher). Protein concentration was measured with Bradford Protein Assay (Bio-Rad). Nuclear extracts (50 μg) were then subjected to 12% SDS-PAGE. Proteins were probed with an antibody specific to CXXC5 (ab106533, Abcam) or PARP1 (9542; Cell Signaling Tech.) followed by a secondary antibody conjugated with horseradish peroxidase (Advansta Inc., San Jose, CA, USA). The HDAC1 antibody (Abcam, ab19845) was used for monitoring the levels of HDAC1, which was used as the loading control. Proteins were visualized with ECL (Advansta) and images were captured with ChemiDoc Imaging System (Bio-Rad). PageRuler Prestained Protein Ladder (Thermo-Fisher) was used as a molecular weight marker.

### Cellular proliferation

To assess the effects of CXXC5 synthesis on cellular growth, cells were plated in 96-well culture plates in phenol red-free medium containing 10% CD-FBS for 48 h. Cells were then transiently transfected with siRNAs as described previously^4^ in the absence (ethanol, 0.01%) or the presence of E2 (10^−8^ M) and/or ICI (10^−6^ M). 48 h after transfection, cells were subjected to counting using a hemocytometer or 3-(4,5-Dimethylthiazol-2-yl)−2,5 diphenyltetrazolium bromide (MTT) assay (Sigma-Aldrich) as described previously^3^.

### Cell cycle analysis

To assess the effects of reduced CXXC5 protein levels on cell cycle phases, cells, 16 ×10^4^/well of a six-well tissue culture plate, were grown in phenol red-free medium containing 10% CD-FBS for 48 h. Cells were then transfected without or with siRNAs in the absence (ethanol, 0.01%) or the presence of 10^−8^ M E2 and grown for additional 48 h. Cells were collected with trypsinization, washed with PBS and pelleted. Cells were then processed for cell cycle distribution as described previously^3,65^. In brief, pelleted cells were gently re-suspended in 100 µl of 2%CD-FBS containing PBS, fixed and permeabilized with ice-cold 70% ethanol overnight. Cells were subsequently incubated with 200 µl of PBS containing propidium iodide (20 µg/ml; Sigma-Aldrich), 200 µg/ml RNase A (Thermo-Fisher) and 0.1% (v/v) Triton X-100 (AppliChem, Germany) for 30 min. Cell cycle analyses were then carried out with flow cytometry (BD Accuri C6 Cytometer; BD Biosciences, San Jose, CA, USA).

### Annexin V assay

To assess whether reduced CXXC5 protein levels cause cell death in the absence or presence of E2, MCF7 cells, 16 × 10^4^ cells/well of a six-well tissue culture plate, were grown in phenol red-free medium containing 10% CD-FBS for 48 h for. Cells were then transfected without or with siRNAs in the absence (ethanol, 0.01%) or the presence of 10^−8^ M E2 and grown for additional 48 h. Cells were subsequently subjected to Annexin V staining, as we described previously^3,65^, according to the manufacturer’s instructions (BioLegend, San Diego, CA, USA). In brief, cells were harvested and washed twice with cold 1X PBS and then re-suspended in 1X binding buffer. APC Annexin V (5 µl) and 7-Amino-Actinomycin (7-ADD) (5 µl) were added to 100 µl of cell suspension containing 10^5^ cells. The cell suspension was gently mixed and incubated for 15 min at room temperature in the dark followed by the addition of 400 µl of 1X binding buffer and subjection to flow cytometry (BD Accuri C6 cytometer, BD Biosciences). Using the experimental approach described here for Annexin V assay, we also carried out WB for the cleavage of Poly(ADP-ribose) Polymerase (PARP) as an indication of apoptosis, with a PARP1-specific antibody (9542, Cell Signaling Tech.) that also detects the cleaved PARP. We also used camptothecin (CPT), a DNA topoisomerase inhibitor, as a control for the induction of cell death. Cells maintained for 48 h in the absence or presence of E2 were subjected to 2 nM CPT for 24 h. Cells were then collected and subjected to Annexin V staining or WB analysis for PARP1 cleavage using the PARP1 antibody.

### Analysis of Gene Expression with nCounter PanCancer Pathway Panel gene expression analysis and RT-qPCR

MCF7 cells in 12-well tissue culture plates (9 ×10^4^ cells/well) were cultured in phenol red-free medium containing 10% CD-FBS medium for 48 h. Cells were then transiently transfected with the HiPerfect transfection reagent (Qiagen) using 10 nM of an siRNA. Forty-eight hours after transfection, cells were treated without (ethanol, 0.01%) as vehicle control or with 10^−8^ M of E2 for 3 hours. For RNA extraction, Quick-RNA MiniPrep (Zymo Research, Irvine, CA, USA) was used as instructed by the manufacturer. The total RNA sample was measured using BioDrop μLITE (Cambridge, United Kingdom). 50 ng RNA/sample was used for processing the nCounter PanCancer Pathway Panel gene expression analysis (NanoString Technologies, Inc. Redwood City, CA, USA). This panel contains 730 different probes together with 40 internal control probes complementary to the corresponding target mRNAs. The fluorescence barcodes specific for a gene are attached to each probe, enabling digital counting of each transcript. Sample preparation was carried out with nCounter PrepStation and microscopy scanning of the barcode signals was carried out with nCounter Digital Analyzer (Nanostring) as instructed by the manufacturer. Quality control, data normalization and differential expression analyses were done using Nanostring nSolver 3.0 Analysis software together with its Advanced Analysis plug-in.

For RT-qPCR, total RNA isolated from MCF7 cells, treated as described for PanCancer Pathway Panel, was used for cDNA synthesis (The RevertAid First Strand cDNA Synthesis Kit, Thermo-Fisher). Based on the results from PanCancer Pathway Panel gene expression analysis, eight genes were selected for the verification of the results observed with PanCancer Pathway Panel with RT-qPCR using gene-specific primers (Supplementary Table S1). RT-qPCR products were detected with SYBR Green on BioRad Connect Real-Time PCR. We used the expression of *RPLP0* for qPCR normalizations.

### Metadata analysis for the clinical relevance of *CXXC5* in breast cancer patients

mRNA expressions of CXXC5 and ERα in patients from METABRIC^69^ and TCGA (https://www.cancer.gov/tcga) databases were downloaded from cBioPortal^70,71^. Protein expression of ERα was downloaded from TCPA database^72^. Survival curves were generated based on median separation and the significance of the difference among groups was determined using the Log-rank test. Comparisons between the two groups were made by a two-tailed t-test. Pearson correlation coefficient was calculated to demonstrate the association between gene or protein expressions of CXXC5 and ERα. Box-plots depict median number and the 25th to 75th quartiles. “ns” denotes “not significant”.

### Methylation profiling

A publically available methylation profiling dataset of MCF-7 cells in response to E2 (GSE132513) was used to analyze the differential methylation of E2-regulated genes that are affected by CXXC5 knockdown. To this end, normalized methylation scores were downloaded from the GEO database, and the ratio of the methylation scores in “E2 stimulated” cells to “E2-deprived” cells were taken. A fold change cut-off of 1.2 was used to determine differentially methylated genes^73,74^. A heatmap of the methylation scores of E2-regulated genes whose expression depends on CXXC5 modulation was drawn using Morpheus software.

### Statistical analysis

Results, with biological replicates as indicated for each experiment, were presented as the mean ± standard error (S.E.) The statistical analyses were performed using one-way ANOVA with Tukey test for post-hoc analysis or two-tailed unpaired t-test with a confidence interval, minimum, of 95%.

## Supplementary information

Supplementary information.
