# Author Correction: CXXC5 as an unmethylated CpG dinucleotide binding protein contributes to estrogen-mediated cellular proliferation

**DOI:** 10.1038/s41598-020-66682-7

**Published:** 2020-06-16

**Authors:** Gamze Ayaz, Negin Razizadeh, Pelin Yaşar, Gizem Kars, Deniz Cansen Kahraman, Özge Saatci, Özgür Şahin, Rengül Çetin-Atalay, Mesut Muyan

**Affiliations:** 1 Department of Biological Sciences, Middle East Technical University, Ankara, 06800 Turkey; 2 Enformatics Institute, Middle East Technical University, Ankara, 06800 Turkey; 3 Cansyl Laboratories, Middle East Technical University, Ankara, 06800 Turkey; 4 Drug Discovery and Biomedical Sciences, College of Pharmacy, University of South Carolina, Columbia, SC 29208 USA; 5 Department of Molecular Biology and Genetics, Bilkent University, Ankara, 06800 Turkey; 6 Present Address: Cancer and Stem Cell Epigenetics Section, Laboratory of Cancer Biology and Genetics, Center for Cancer Research, National Cancer Institute, National Institutes of Health, Bethesda, MD 20892 USA

Correction to: *Scientific Reports* 10.1038/s41598-020-62912-0, published online 06 April 2020

The version of this Article previously published contained lower resolution images for Figures 1 and 3. This has now been corrected in the PDF and HTML versions of the paper.

