# Supplementary material for: CXXC5 as an unmethylated CpG dinucleotide binding protein contributes to estrogen-mediated cellular proliferation
Source: Sci Rep. 2020 Apr 6;10:5971. doi: 10.1038/s41598-020-62912-0 (PMC7136269; doi:10.1038/s41598-020-62912-0)
Supplement: Supplementary file 1 — Supplementary information. [file 41598_2020_62912_MOESM1_ESM.pdf]

SUPPLEMENTARY INFORMATION

CXXC5 as an unmethylated CpG dinucleotide binding protein contributes to estrogen-mediated cellular proliferation

Gamze Ayaz[1,6], Negin Razizadeh[1], Pelin Yaşar[1], Gizem Kars[1], Deniz Cansen Kahraman[2,3], Özge Saatci[4], Özgür Şahin[4,5], Rengül Çetin-Atalay[2,3], and Mesut Muyan[1,3*]

[1]Department of Biological Sciences, Middle East Technical University, Ankara 06800, Turkey; [2]Enformatics Institute & [3]Cansyl Laboratories, Middle East Technical University, Ankara 06800, Turkey; [4]Drug Discovery and Biomedical Sciences, College of Pharmacy, University of South Carolina, SC, USA; [5]Department of Molecular Biology and Genetics, Bilkent University, Ankara 06800, Turkey

[*]Corresponding Author: mmuyan@metu.edu.tr

[6]Current Address: Cancer and Stem Cell Epigenetics Section, Laboratory of Cancer Biology and Genetics, Center for Cancer Research, National Cancer Institute, National Institutes of Health, Bethesda, MD, 20892, USA

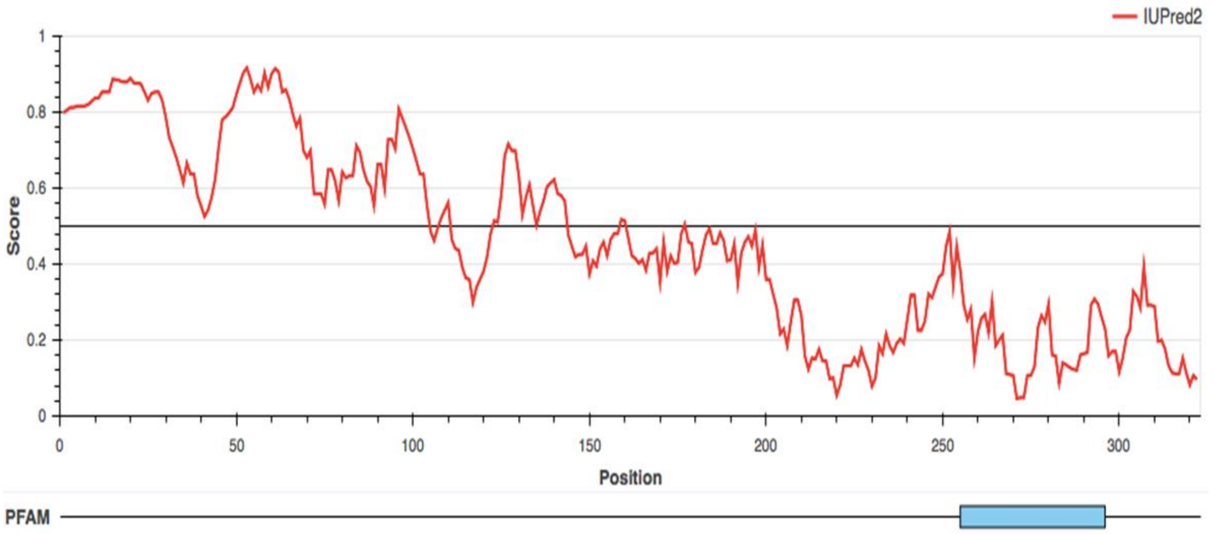

Supplementary Fig. S1. Prediction of intrinsically unstructured regions of CXXC5 protein by the IUPred web server (https://iupred2a.elte.hu). The prediction of intrinsically unstructured regions from amino acid sequences is dependent on the estimation of the energy content of total pairwise inter-residue interactions. The score ranges from 0 (complete order) to 1 (complete disorder). A score above 0.5 is accepted as intrinsically unstructured regions, as seen in the amino-terminus of CXXC5. The blue box indicates the CXXC domain.

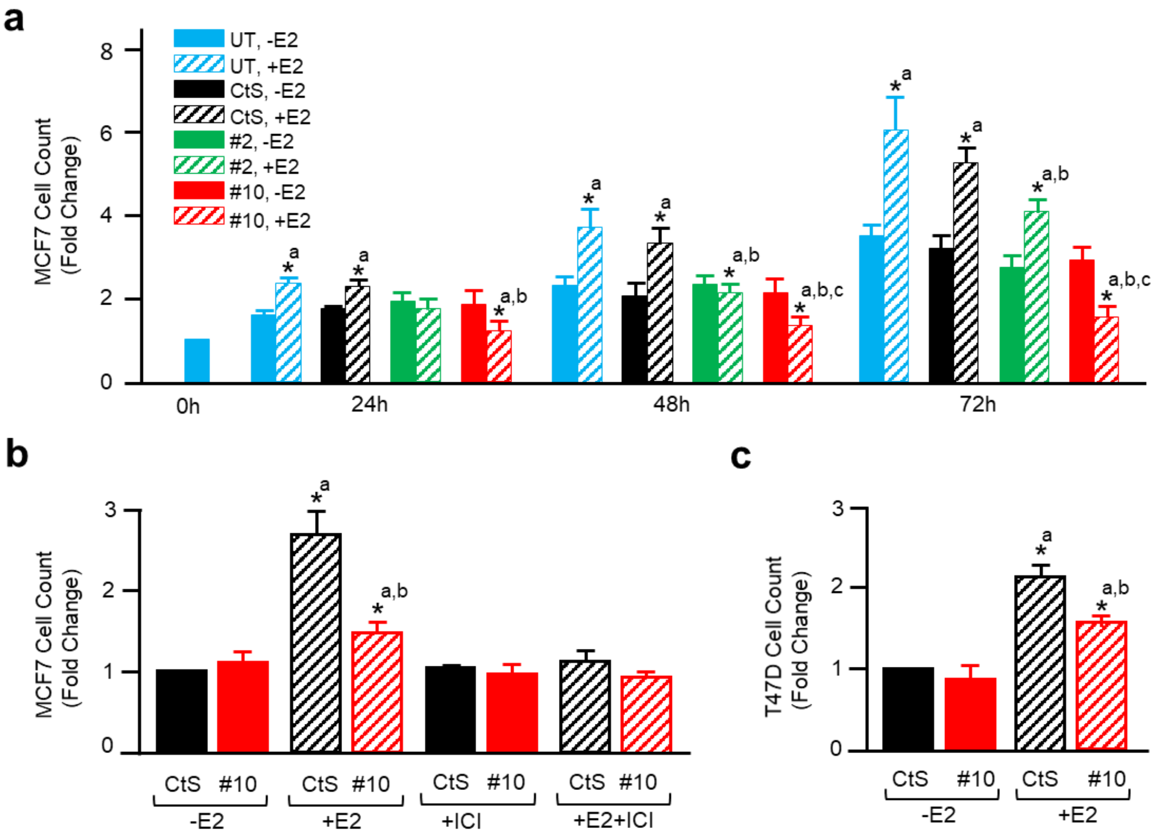

Supplementary Fig. S2. Ligand effects on the growth of cells. Effects of siRNAs specific to CXXC5 on cellular growth. **(a)** MCF7 cells grown in 10% CD-FBS containing medium for 48h were transiently transfected without (UT) or with CtS, siRNA#2 or siRNA#10 in the absence (0.01% ethanol) or the presence of 10^{-8} M E2 for 24h, 48h or 72h. Cells were then subjected to cell counting using hemocytometer or MTT assay. Results, which are the means ± S.E. of three independent determinations, depict fold change in cellular growth compared with those observed with cells at time 0 in the absence of E2, which is set to 1. The asterisk indicates significant change, the superscript "a": significantly different from the corresponding ethanol-treated group (-E2); superscript "b": significantly different from CtS transfected cells in the presence of E2; superscript "c": significantly different from siRNA#2 transfected cells in the presence of E2. **(b)** MCF7 or **(c)** T47D cells grown in 10% CD-FBS containing medium for 48h were transiently transfected with CtS or siRNA#10 in the absence (0.01% ethanol) or the presence of 10^{-8} M E2. MCF7 cells were also transfected in the presence of 10^{-6} M ICI (+ICI) or of 10^{-8} M E2 and 10^{-6} M ICI (E2+ICI) for 48h. Cells were then subjected to cell counting using a hemocytometer. Results, which are the means ± S.E. of three independent determinations, depict fold change in cellular growth compared with cells transfected with CtS in the absence of E2, which is set to 1. Asterisk with superscript "a" indicates significant difference from CtS transfected cells in the absence of E2; whereas superscript "b" depicts a significant difference from transfected cells in the presence of E2.

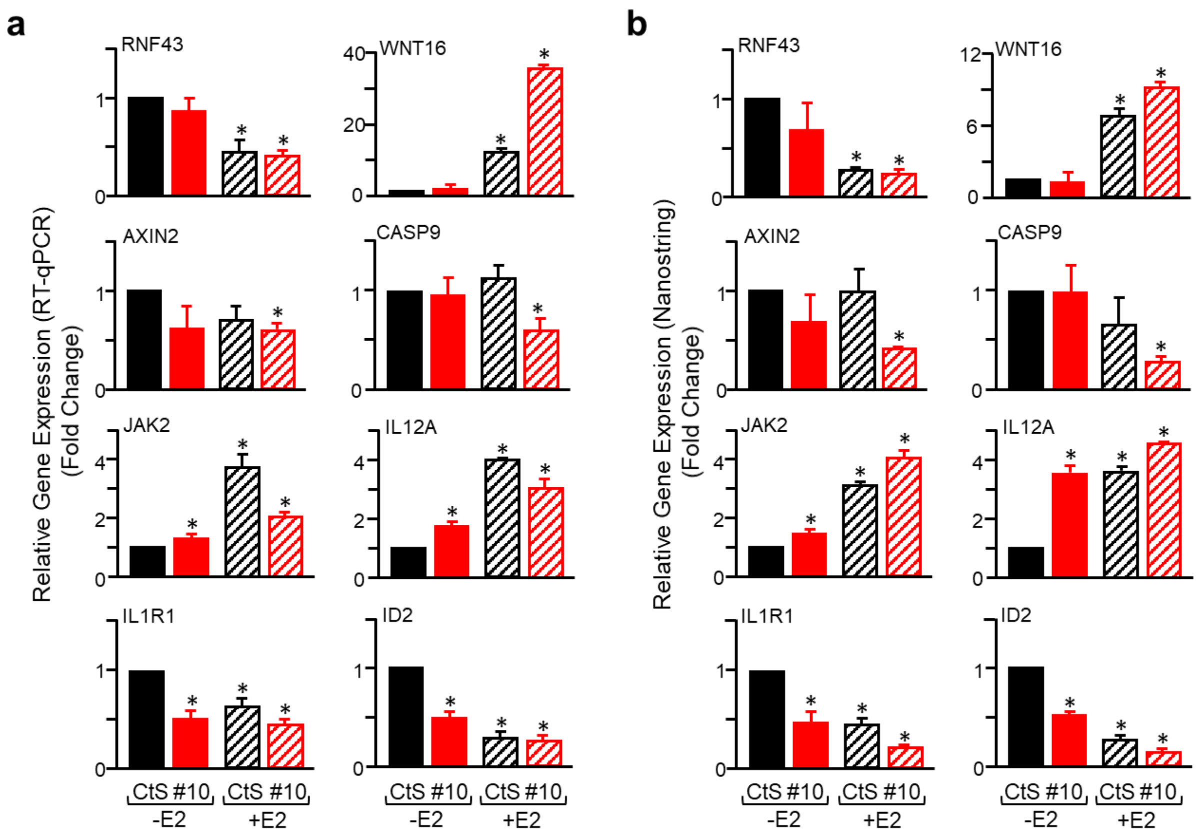

Supplementary Fig. S3. Analysis of gene expressions using RT-qPCR. (**a, b**) MCF7 cells grown in 12-well tissue culture plates (9 x 10^4 cells/well) in 10% CD-FBS medium for 48h were transiently transfected using 10 nM of CtS or siRNA#10. Forty-eight hours after transfection, cells were treated without (-E2; ethanol, 0.01% as vehicle control) or with 10^{-8} M of E2 (+E2) for 3 hours. **(a)** Total RNA was subjected to cDNA conversion followed by qPCR using a primer set specific to a selected gene. Results, which are the mean ± S.E. of three independent determinations, depict fold changes in mRNA levels compared with those observed in cells transiently transfected with CtS in the absence of E2, which is set to 1. The asterisk indicates a significant change. **(b)** Shown is also the gene expression analysis of PanCancer Pathway Panel for comparisons.

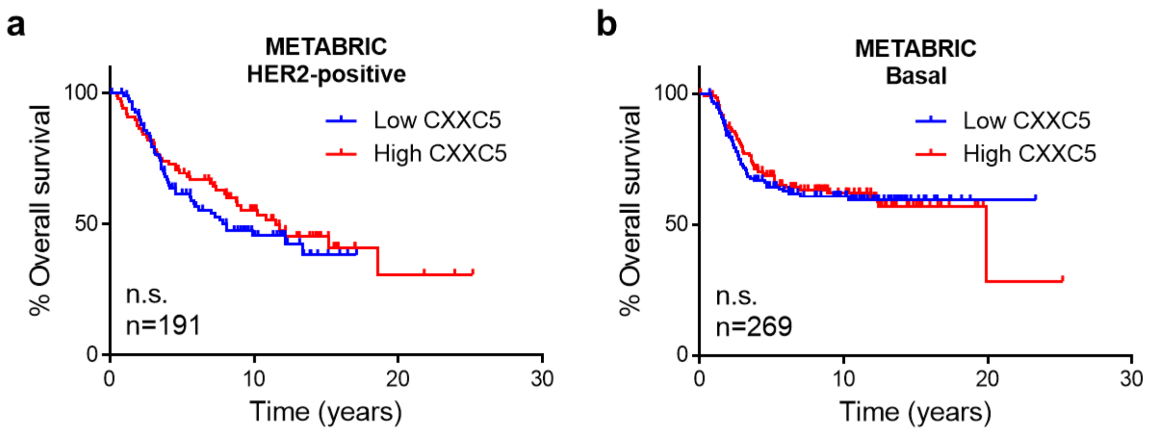

Supplementary Fig. S4. The effect of CXXC5 expression on the clinical outcome of HER2+ and Basal-like subtypes. Kaplan Meier survival analysis of **(a)** HER2+ or **(b)** Basal-like breast cancer patients based on *CXXC5* expression separated from median.

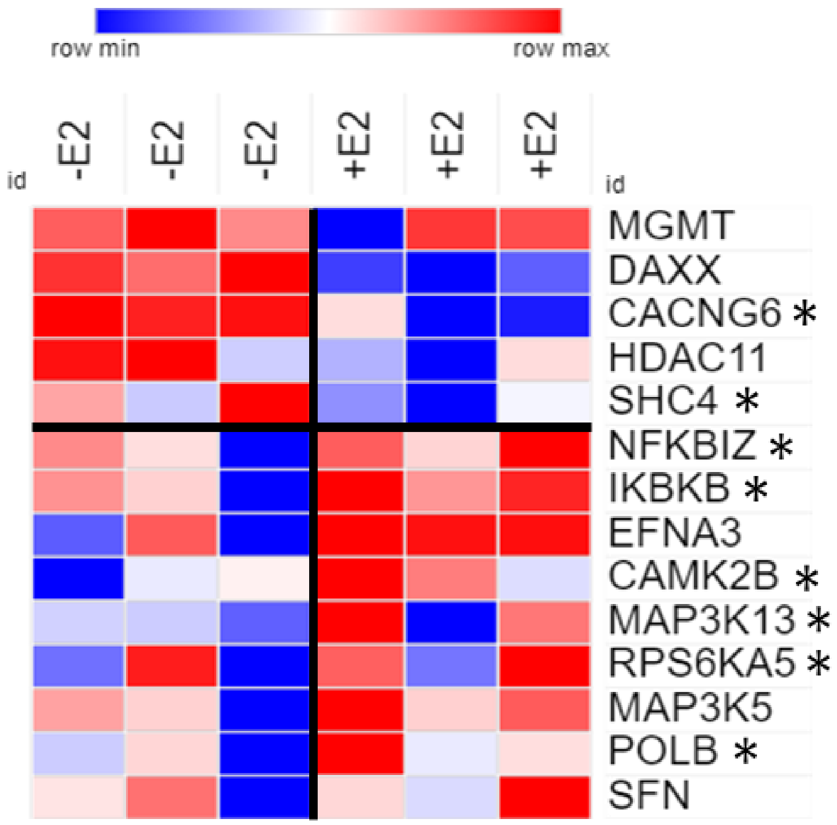

Supplementary Fig. S5. Methylation profiling: A methylation profiling dataset of MCF7 cells under E2 stimulation (GSE132513) was used to analyze the differential methylation of E2-regulated genes that are affected by CXXC5 knockdown. To this end, normalized methylation scores were downloaded from the GEO database, and the ratio of methylation scores in "E2 stimulated" cells to "E2-deprived" cells were taken. A fold change cut-off of 1.2, in the absence (-E2) or presence (+E2) of E2 in three biological replicates, was used to determine differentially methylated genes. A heatmap of the methylation scores, with increased (red) and decreased levels (blue), of E2-regulated genes whose expression depends on CXXC5 modulation was generated using Morpheus software (https://software.broadinstitute.org/morpheus/). Asterisks indicate changes in the promoter regions.

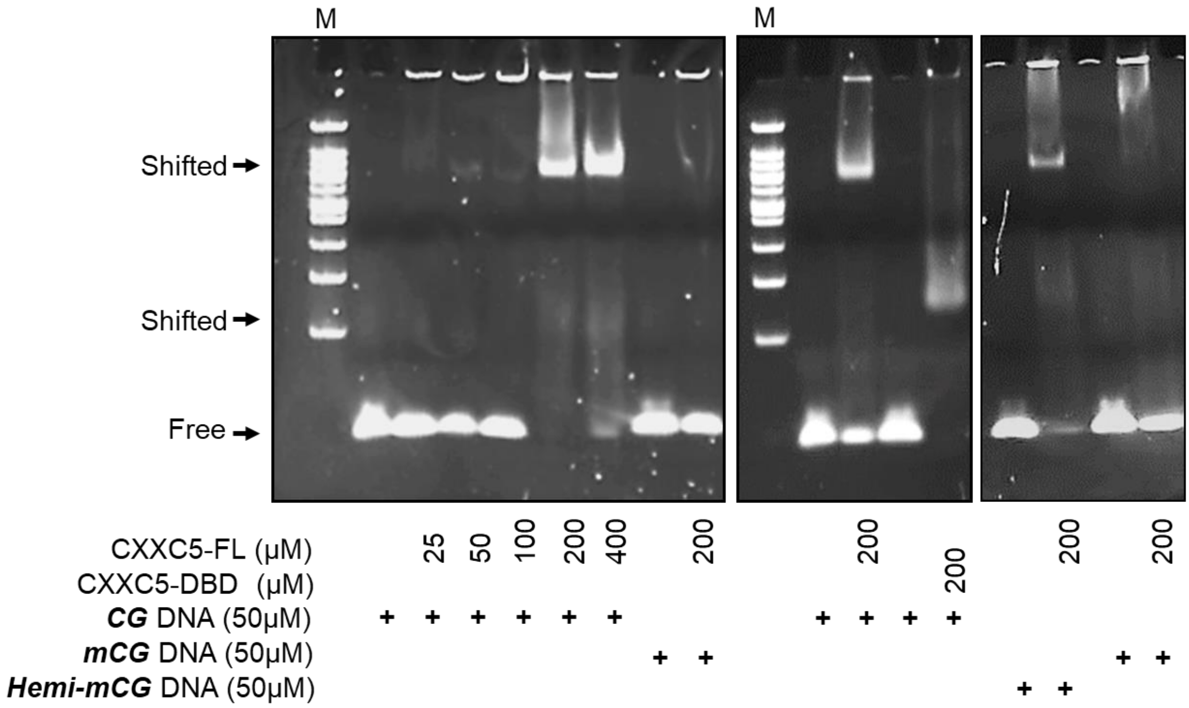

Supplementary Fig. S6. Electrophoretic mobility shift (EMSA). To assess the ability of the recombinant FL-CXXC5 or its CXXC domain (CXXC-D), 50 μM double-stranded DNA fragment that bears a central unmethylated ***CG*** (5'-GTGATAC***CG***GATCAGT-3' or methylated (**mCG**) CpG dinucleotides was mixed with FL-CXXC5 at 1:0.5, 1:1, 1:2, 1:4 and 1:8 molar ratios or with CCCX-D at 1:4 molar ratios. FL-CXXC5 was also mixed with DNA fragment bearing hemimethylated DNA fragment (the upper strand: 5'-GTGATAC***CG***GATCAGT-3'; the lower strand: 5'-GTGATAC***mCG***GATCAGT-3') at 1:4 molar ratios. Samples were run onto 5% native TBE gel, stained and visualized with UV spectrometry. "M" represents the molecular marker. "Free" denotes unbound DNA; "Shifted" indicates DNA bound protein. A representative experiment performed with two independent times is shown.

Supplementary Table S1. Nucleotide sequences of primers for RT-qPCR and siRNAs. Shown are the nucleotide sequences of primers for selected genes and of siRNAs specific for CXXC5 or a scrambled siRNA as control (CtS).

Primers for RT-qPCR, 5' to 3'

	**Forward**	**Reverse**
**CXXC5**	CGGTGGACAAAAGCAACCCTAC	CGCTTCAGCATCTCTGTGGACT
**RPLP0**	GGAGAAACTGCTGCCTCATA	GGAAAAAGGAGGTCTTCTCG
**AXIN2**	CAAACTTTCGCCAACCGTGGTTG	GGTGCAAAGACATAGCCAGAACC
**CASP9**	GTTTGAGGACCTTCGACCAGCT	CAACGTACCAGGAGCCACTCTT
**ID2**	TTGTCAGCCTGCATCACCAGAG	AGCCACACAGTGCTTTGCTGTC
**IL12A**	TGCCTTCACCACTCCCAAAACC	CAATCTCTTCAGAAGTGCAAGGG
**IL1R1**	GTGCTTTGGTACAGGGATTCCTG	CACAGTCAGAGGTAGACCCTTC
**JAK2**	CCAGATGGAAACTGTTCGCTCAG	GAGGTTGGTACATCAGAAACACC
**RNF43**	GGTTACATCAGCATCGGACTTGC	ATGCTGGCGAATGAGGTGGAGT
**WNT16**	TCGGAAACACCACGGGCAAAGA	GCGGCAGTCTACTGACATCAAC

siRNA Sequence, 5' to 3'

**siRNA#2**	CAGCAGTTGTAGGAATCGAAA
**siRNA#10**	TCAGATTTGCAAATTCAGAAA
**CtS**	TTCTCCGAACGTGTCACGT

Supplementary Table S2. Gene expression analysis using PanCancer Pathway Panel. MCF7 cells grown in 12-well tissue culture plates (9 x 10^4 cells/well) in 10% CD-FBS medium for 48h were transiently transfected using 10 nM of CtS or siRNA#10. Forty-eight hours after transfection, cells were treated without (ethanol, 0.01% as vehicle control) or with 10^{-8} M of E2 for 3 hours. Total RNA (50 ng) was subjected to the nCounter PanCancer Pathway Panel gene expression analysis with nCounter Digital Analyzer. Quality control, data normalization and differential expression analyses were carried out using Nanostring nSolverTM 3.0 Analysis software together with its Advanced Analysis plug-in. Results from each treatment group normalized to the results obtained with indicated groups in the absence (-E2) or the presence of E2 (+E2) are shown with increasing and decreasing (-) levels and are the mean of fold change in log2 scale from three independent determinations.

Group 2/ Group1 (#10 and -E2/CtS and -E2)				Group 3/ Group1 (CtS and +E2/CtS and -E2)				Group 4/ Group2 (#10 and +E2/#10 and -E2)		
**Gene**	**log2**	**P-value**		**Gene**	**log2**	**P-value**		**Gene**	**log2**	**P-value**
APC	-0.512	0.0166		ALKBH2	0.433	0.039		ALKBH3	0.35	0.0346
ASXL1	-0.252	0.0473		ARID1A	-0.26	0.0254		ARID1B	0.236	0.00484
AXIN1	0.206	0.0289		ARID1B	0.389	0.000219		ATR	0.478	0.00876
BAX	0.37	0.00402		AXIN1	0.54	0.000115		AXIN1	0.27	0.00828
BID	0.354	0.013		BCL2	0.822	0.00023		BCL2	0.625	0.00136
BIRC3	1.39	0.0183		BCOR	-0.372	0.0279		BCOR	-0.332	0.0437
BMP7	0.385	0.043		BIRC3	1.49	0.0128		BIRC3	1.7	0.000848
CASP7	-0.39	0.0211		CACNG6	1.1	0.0187		BMP7	-0.573	0.00723
CCND1	0.538	0.00205		CAMK2B	-0.515	0.0405		CASP7	0.494	0.00676
CCNE1	0.247	0.0168		CASP7	0.642	0.00154		CASP8	0.236	0.0423
CDC25B	-0.424	0.00237		CCND1	0.93	5.55E-05		CCND1	0.561	0.00161
DAXX	0.21	0.016		CDC25A	0.467	0.0375		CDK6	-0.69	0.0328
DDB2	0.724	0.00158		CDKN2B	-0.52	0.0101		CDKN2B	-0.898	0.000525
DKK1	0.341	0.00451		DAXX	0.206	0.0176		CEBPA	-0.301	0.008
FANCF	0.189	0.0193		DDIT4	-0.47	0.000488		CIC	-0.553	0.0101
FAS	1.75	0.0026		DUSP2	-0.293	0.0376		CREB5	0.373	0.0293
FBXW7	0.335	0.0226		DUSP4	0.452	0.0134		DDIT4	-0.229	0.0252
FOSL1	0.683	0.0479		E2F5	0.554	0.00228		DKK1	-0.281	0.0123
FZD8	-0.803	0.00262		EFNA1	-0.531	0.000919		DUSP2	-0.341	0.02
GADD45A	0.425	0.0335		EFNA3	-0.375	0.0402		DUSP4	0.443	0.0152
GRB2	0.372	8.61E-05		ETS2	0.351	0.0268		EFNA1	-0.429	0.00331
HDAC10	0.408	0.0382		FGF18	1.62	0.00232		ENDOG	0.12	0.0493
ID1	-0.492	0.0322		FOSL1	0.688	0.0467		ERBB2	-0.381	0.00648

ID2	-0.455	0.0246		FZD8	-0.657	0.00784		FUT8	-0.335	0.0346
IDH1	-0.3	0.0309		GLI3	-0.39	0.0217		FZD7	0.769	0.036
IL12A	1.7	0.0464		H3F3C	-0.127	0.0135		FZD8	0.452	0.0419
IL1R1	-1.02	0.0183		HDAC11	0.702	0.00147		GATA3	-0.355	0.00446
ITGA2	-0.397	0.0159		ID1	-0.709	0.00582		GLI3	-0.368	0.0277
JAK2	0.8	0.00085		ID2	-1.02	0.000281		GNG12	0.0772	0.00856
KAT2B	0.308	0.036		IKBKB	-0.632	0.0238		HDAC5	-0.341	0.0485
KITLG	-0.361	0.0173		IL12A	1.77	0.04		HHEX	-0.607	0.00424
LRP2	-1.16	0.0251		IL1R1	-1.08	0.0135		ID1	-0.885	0.00169
LTBP1	0.23	0.00211		IL24	1.47	0.00797		ID2	-1.17	0.000128
MAP2K2	0.163	0.000892		IL8	0.789	0.0251		ID4	-0.615	0.0296
MAP2K4	0.352	0.0107		INHBB	0.543	0.00255		IL1R1	-1.47	0.0027
MAP3K1	0.175	0.0489		IRS1	0.596	0.000836		IL1RAP	0.876	0.00602
MAP3K13	-1.04	0.0133		JAK1	0.311	0.0267		IL24	1.31	0.0125
MAP3K14	0.528	0.017		JAK2	1.8	2.03E-06		IRS1	0.335	0.0194
MAPK3	-0.173	0.00155		KITLG	-0.707	0.000389		ITGB6	-0.57	0.00464
MAPK8IP2	0.86	0.00387		KLF4	0.387	0.00959		ITGB8	-0.86	0.00549
MAPT	0.293	0.0482		LIFR	-0.587	0.0331		JAG2	-0.389	0.0276
MDM2	-0.345	0.00286		MAP3K1	0.336	0.00211		JAK1	0.297	0.0323
MNAT1	-0.349	0.0122		MAP3K13	-0.73	0.0466		JAK2	1.19	2.64E-05
MTOR	-0.401	0.0101		MAP3K5	-0.555	0.0214		KAT2B	-0.403	0.011
MYC	0.376	0.000109		MAPK3	-0.107	0.0195		KITLG	-0.366	0.0166
MYD88	0.257	0.024		MGMT	0.0973	0.0368		KRAS	-0.235	0.0348
NBN	0.153	0.0226		MYB	0.722	0.00413		LAMA3	0.769	0.0408
NF2	0.186	0.0159		MYC	0.459	2.62E-05		LTBP1	-0.148	0.0211
NFKB1	0.369	0.0258		NFKBIZ	-0.52	0.00295		MAP3K1	0.378	0.00101
NFKBIA	0.288	0.0435		PBRM1	0.177	0.0201		MYC	0.17	0.013
NR4A3	-0.814	0.0435		PDGFA	-0.441	0.0368		NBN	0.128	0.0468
NUMBL	0.279	0.0449		PIK3R1	0.219	0.00896		NOTCH3	-0.343	0.0376
PIK3CA	-0.388	0.0256		PIK3R3	-0.538	0.000465		PBRM1	-0.221	0.00682
PIK3R3	-0.244	0.033		PLA2G3	-1.04	0.0457		PDGFA	-0.484	0.0254
PLCB4	-0.438	0.0437		POLB	-0.3	0.0236		PIK3R1	0.16	0.0369
POLD4	-0.215	0.0354		POLR2D	0.219	0.00208		PIK3R3	-0.478	0.000984
POLR2J	-0.241	0.0171		PTEN	-0.133	0.0364		PLD1	-1.42	0.00968
PPP3CC	0.706	0.0029		RASGRP1	1.49	1.33E-05		POLR2D	0.25	0.000937
PPP3R1	-0.277	0.000149		RET	1.16	4.16E-05		PPP2CB	-0.189	0.0201
PRKAR2A	-0.924	7.77E-05		RNF43	-1.98	0.00219		PPP3CB	-0.295	0.00574
PRKAR2B	0.36	0.0155		RPS6KA5	-0.35	0.0494		PTCH1	-1.13	0.0245
RAC3	0.213	0.0498		SETBP1	1.73	0.000646		PTEN	-0.233	0.00234
RAD50	-0.368	0.00237		SFN	-0.186	0.0368		RAD21	-0.165	0.0417
RASGRP1	0.842	0.000816		SHC4	1.05	0.0218		RASGRP1	0.616	0.00336
RET	0.406	0.0255		SMC1A	-0.254	0.0392		RET	1.18	2.79E-05
RPS6KA5	-0.445	0.0186		SOCS3	0.247	0.0125		RNF43	-1.07	0.0441
SETBP1	1.26	0.00429		SPRY1	-0.488	0.0307		SETBP1	0.864	0.027

SHC4	1.3	0.00764		STK11	0.39	0.000905		SIX1	-0.777	0.0491
SKP2	-0.502	0.019		TBL1XR1	-0.299	0.016		SOCS2	-0.915	0.00713
SMARCB1	0.161	0.0179		TET2	0.525	0.000271		SOCS3	-0.303	0.00434
SOCS3	0.321	0.00312		TGFB3	-0.531	0.0349		SOS2	-0.718	0.000697
STAG2	-0.396	0.002		TIAM1	0.522	0.00472		SPRY1	-0.496	0.0287
STK11	0.214	0.0227		VEGFA	0.53	1.47E-05		STK11	0.318	0.00308
SYK	-0.258	0.0496		WEE1	-0.376	0.0176		SUV39H2	0.273	0.0338
TLR2	0.601	0.00615		WNT16	3.87	0.000146		TBL1XR1	-0.356	0.00669
TNFRSF10A	0.427	0.025						TCF3	-0.177	0.0379
U2AF1	0.193	0.0103						TCF7L1	0.693	0.0393
VEGFA	0.242	0.00286						TET2	0.521	0.000288
VHL	0.167	0.0256						TGFB2	-0.979	0.0124
WHSC1	-0.254	0.00952						TGFB3	-0.475	0.048
WHSC1L1	-0.23	0.00977						TIAM1	0.715	0.000722
								UBE2T	0.637	0.0103
								VEGFA	0.548	1.14E-05
								VEGFC	-0.593	0.0374
								WEE1	-0.418	0.0111
								WNT16	3.76	0.000179
								WNT7B	-0.562	0.00243
								ZIC2	0.442	0.0308
